# 3D‐Epigenomic Regulation of Gene Transcription in Hepatocellular Carcinoma

**DOI:** 10.1002/ggn2.202100010

**Published:** 2022-06-29

**Authors:** Yuliang Feng, Ping Wang, Liuyang Cai, Meixiao Zhan, Fan He, Jiahui Wang, Yong Li, Eva Gega, Wei Zhang, Wei Zhao, Yongjie Xin, Xudong Chen, Yijun Ruan, Ligong Lu

**Affiliations:** ^1^ Zhuhai Precision Medical Center Zhuhai People's Hospital Zhuhai Hospital Affiliated with Jinan University Zhuhai Guangdong 519000 P. R. China; ^2^ The Jackson Laboratory for Genomic Medicine Farmington CT 06032 USA; ^3^ Department of Interventional Radiology Shenzhen People's Hospital (The Second Clinical Medical College, Jinan University; The First Affiliated Hospital, Southern University of Science and Technology) Shenzhen Guangdong 518020 P. R. China

**Keywords:** 3D genome, 3D‐epigeome, ChIA‐PET, chromatin topology, hepatocellular carcinoma, transcription regulation

## Abstract

The fundamental cause of transcription dysregulation in hepatocellular carcinoma (HCC) remains elusive. To investigate the underlying mechanisms, comprehensive 3D‐epigenomic analyses are performed in cellular models of THLE2 (a normal hepatocytes cell line) and HepG2 (a hepatocellular carcinoma cell line) using integrative approaches for chromatin topology, genomic and epigenomic variation, and transcriptional output. Comparing the 3D‐epigenomes in THLE2 and HepG2 reveal that most HCC‐associated genes are organized in complex chromatin interactions mediated by RNA polymerase II (RNAPII). Incorporation of genome‐wide association studies (GWAS) data enables the identification of non‐coding genetic variants that are enriched in distal enhancers connecting to the promoters of HCC‐associated genes via long‐range chromatin interactions, highlighting their functional roles. Interestingly, CTCF binding and looping proximal to HCC‐associated genes appear to form chromatin architectures that overarch RNAPII‐mediated chromatin interactions. It is further demonstrated that epigenetic variants by DNA hypomethylation at a subset of CTCF motifs proximal to HCC‐associated genes can modify chromatin topological configuration, which in turn alter RNAPII‐mediated chromatin interactions and lead to dysregulation of transcription. Together, the 3D‐epigenomic analyses provide novel insights of multifaceted interplays involving genetics, epigenetics, and chromatin topology in HCC cells.

## Graphical abstract

THLE2 cells of normal liver hepatocytes and HepG2 cells of hepatocellular carcinoma (HCC) are comprehensively analyzed via integrated approaches including ChIA‐PET for 3D‐genome mapping and RNA‐seq, ChIP‐seq, and whole genome bisulfite sequencing for epigenome mapping, thus the 3D‐epigenome. The comparative analyses of the 3D‐epigenomes for normal hepatocytes and HCC cells reveal HCC‐associated genes for suppressing normal functions (HCC‐suppressed genes) in hepatocytes and activating new properties (HCC‐specific genes) in HCC.

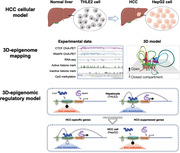

## 1 Introduction

Hepatocellular carcinoma (HCC) is the sixth most common cancer in the world and the second leading cause of cancer‐related mortality in men.^[^ ^1^ ^]^ In spite of recent progress in surgical resection/transplantation, radio‐frequent ablation, targeted drug therapy, and immunotherapy (e.g., anti‐PD‐1/PD‐L1 agents), the overall prognosis of HCC is still dismal.^[^ ^1^ ^]^ Thus, there is a pressing need for the development of novel therapies for HCC, which depends on a better mechanistic understanding of its pathogenesis.

Recently, large‐scale RNA sequencing efforts from the International Cancer Genome Consortium (ICGC) and The Cancer Genome Atlas (TCGA) have revealed that gene expression is pervasively dysregulated in HCC.^[^ ^2^, ^3^ ^]^ However, the underlying mechanism of transcription regulation remains elusive. In the past decade, *cis*‐regulatory elements (e.g., promoters and enhancers) and trans‐regulatory factors (e.g., transcription factors TFs) have been recognized as essential for the establishment of specific patterns of gene expression.^[^ ^4^ ^]^ In addition, it is becoming increasingly evident that distinct transcriptional profiles responsible for cell‐specific identity not only rely on the linear genome landscape, but also the 3D genome architecture that orchestrates the interaction of distal cis‐regulatory element mediated by trans‐acting TFs.^[^ ^5^, ^6^ ^]^ Disorganization of 3D genome architecture has been implicated in the development of gastrointestinal stromal tumors (GIST),^[^ ^7^ ^]^ T cell acute lymphoblastic leukemia (T‐ALL),^[^ ^8^, ^9^ ^]^ glioma,^[^ ^10^ ^]^ prostate cancer,^[^ ^11^, ^12^ ^]^ and multiple myeloma.^[^ ^13^ ^]^ Nevertheless, the characteristics of 3D genome configuration with related epigenomic features, the extent and function of interactome, and their roles in establishing and maintaining HCC‐specific transcriptional programs have not been fully explored.

In this study, we report our integrated 3D‐epigenomic analyses to interrogate the underlying topological and epigenomic mechanisms that control gene transcription specific in HCC cells. Our results provide novel insights into the interplays of genetic and epigenetic elements that alter 3D chromatin architecture and lead to HCC‐associated transcription regulation.

## 2 Results

### 2.1 Study Design

**Figure 1:**
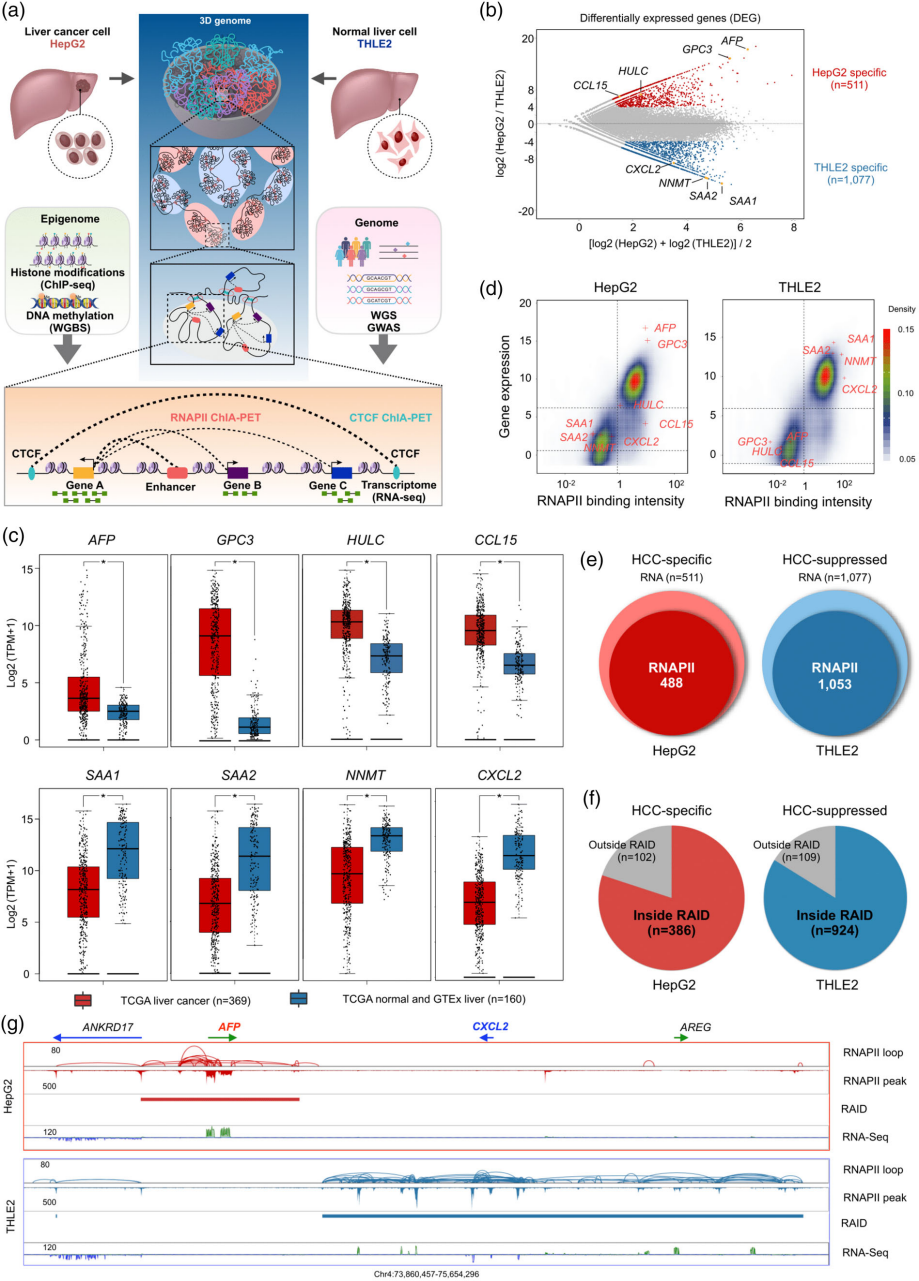
Identification of HCC‐specific genes using RNA‐seq and RNAPII ChIA‐PET data. A) Schematic outline of project design. In vitro cellular models of HepG2 (HCC) and THLE2 (normal hepatocyte) were used in studying the mechanism of transcription dysregulation in liver cancer via integrated 3D‐epigenomic approaches including RNA‐seq, histone ChIP‐seq, ChIA‐PET, WGS/GWAS, and WGBS. B) MA plot showing differentially expressed genes via RNA‐seq data in HepG2 and THLE2 cells. Up‐regulated genes in HepG2 and THLE2 cells are marked in red and blue, respectively. The top and bottom boundaries represent ± 4‐fold change of Log2 (HepG2/THLE2) in gene expression. Representative HepG2‐specific and THLE2‐specific genes are highlighted in orange dots with annotation. C) Boxplot of gene expression for HCC marker genes (*AFP*, *GPC3*, *CCL15*, *HULC*) and normal liver marker genes (*SAA1*, *SAA2*, *NNMT*, *CXCL2*) using TCGA (liver cancer) and GTEx (normal liver tissue) RNA‐seq data. TPM, transcripts per million. * *p*< 0.01 (one‐way ANOVA test). D) Contour plot showing the correlation of RNAPII binding (*x*‐axis) and gene expression (*y*‐axis) in HepG2 and THLE2 cells. Corresponding marker genes are indicated by red cross (+). E) Venn diagram depicting the numbers of cell‐specific genes with overlapping RNAPII binding peaks at gene promoters (TSS) in HepG2 (red) and THLE2 cells (blue). F) Distribution of HCC‐specific genes in HepG2 (red) and HCC‐suppressed genes in THLE2 (blue) inside and outside of RAID (RNAPII‐associated interaction domain), respectively. G) BASIC browser screenshot of a genomic segment (chr4:73860457–75654296) showing HCC‐specific gene (*AFP*) within RAID in HepG2 cells and HCC‐suppressed genes (*CXCL2* and *AREG*) within RAID in THLE2 cells.

To investigate the underlying topological mechanisms that control the gene transcription programs specific in HCC, we used HepG2 cells as a representative for HCC cells and THLE2 cells as normal hepatocytes. We applied an integrated epigenomic and 3D genome mapping methods to interrogate these two cellular systems. Specifically, we performed experiments for comparative analysis on transcriptome (RNA‐seq), epigenome (ChIP‐seq of histone marks, whole‐genome bisulfate sequencing), and 3D genome mapping (Chromatin Interaction Analysis by Paired‐End Tag Sequencing, ChIA‐PET). Considering CCCTC‐binding zinc finger protein (CTCF) as a major chromatin architectural protein^[^ ^14^, ^15^, ^16^, ^17^, ^18^ ^]^ and RNA polymerase II (RNAPII) involved in most gene transcription,^[^ ^19^, ^20^ ^]^ we specifically performed RNAPII ChIA‐PET experiments to map chromatin interactions between promoters and enhancers, and CTCF ChIA‐PET experiments to map chromatin folding architectures. Furthermore, to examine the impact in HCC by genetic and epigenetic variants, we incorporated GWAS^[^ ^21^ ^]^ and TCGA data^[^ ^2^ ^]^ along with epigenomic profiling data generated in this study for comparative analyses (**Figure** **1**A, Table S1, Supporting Information).

### 2.2 Identification of HCC‐Associated Genes Using RNA‐seq and RNAPII ChIA‐PET Data

To identify and characterize HCC‐associated genes, we generated high quality RNA‐seq and RNAPII ChIA‐PET data for HepG2 and THLE2 cells (Table S1, Figure S1A,B, Supporting Information). Comparing gene expression data between HepG2 and THLE2 cells for differentially expressed genes, we identified 511 genes that are significantly up‐regulated in HepG2 cells and 1077 genes that are highly expressed in THLE2 cells (Figure 1B, Figure S1C, Supporting Information). A number of known HCC marker genes (*AFP*, *GPC3*, *CCL15*, *HULC*, Table S2, Supporting Information) are indeed found highly expressed in HepG2 cells, while the expression of several normal liver marker genes (*SAA1*, *SAA2*, *NNMT*, *CXCL2*, Table S2, Supporting Information) are significantly lower in HepG2 cells and higher in THLE2 cells. We further observed that these genes recapitulate the differential transcriptome in liver cancer patients and paired paratumor or normal liver tissues in TCGA/GTEx adatasets (Figure 1C). Moreover, gene expression breadth analysis including 112 ENCODE cells/tissues RNA‐seq data sets (Table S3, Supporting Information) indicated that this set of up‐regulated genes in HepG2 were mostly cell‐specific (Figure S1D, Supporting Information), implying the importance of HepG2‐specific genes in HCC identity (thereafter HCC‐specific genes); whereas most of the genes showing low expression in HepG2 and high in THLE2 were commonly expressed in many cells/tissues, implying that some constitutive functions shared in many tissues might be suppressed in HCC cells (thereafter HCC‐suppressed genes). Collectively, we refer these two groups of genes are HCC‐associated genes.

As RNAPII is the key enzyme in transcription of protein coding genes and non‐coding RNAs, RNAPII is often associated with active genes as measured by RNAPII ChIA‐PET.^[^ ^14^, ^20^, ^22^ ^]^ Each ChIA‐PET experiment provides two genomic datasets—the protein factor binding sites and the chromatin interactions between the binding sites.^[^ ^23^ ^]^ To validate and further refine differential gene expression data, we associated the RNA‐seq data with the RNAPII binding intensity from the RNAPII ChIA‐PET data of HepG2 and THLE2 cells (Table S1, Supporting Information), showing that the genes with high RNAPII binding intensity are also concomitant with high expression in HepG2 and THLE2 cells, respectively (Figure 1D). In addition, we found that 488 (95%) out of the 511 HCC‐specific genes and 1053 (98%) out of 1077 HCC‐suppressed genes have RNAPII binding to the promoters in HepG2 and THLE2 cells, respectively (Figure 1E). As expected, the RNAPII binding intensities in the loci of HCC‐specific genes are significantly higher than in the loci of HCC‐suppressed genes in HepG2 cells (Figure S1E, Supporting Information).

Furthermore, RNAPII‐associated chromatin interactions revealed spatial proximity contacts of transcriptional elements such as distal enhancers and target gene promoters that are linearly in long genomic distance as shown in ChIA‐PET data. As RNAPII ChIA‐PET data often interconnect with multiple active gene promoters and distal enhancers (multi‐gene complex) as a topological mechanism for co‐transcription‐regulation^[^ ^20^ ^]^ in the context of RNAPII‐associated interaction domains (RAIDs), we further characterize HCC‐associated genes according to their participation in RAIDs. We found that most of HCC‐specific genes (386/488 = 79%) and HCC‐suppressed genes (924/1053 = 88%) are located within RAIDs in HepG2 and THLE2 cells, respectively (Figure 1F). For example, the HCC marker gene *AFP* is specifically and highly expressed in HepG2 cells, and it is wrapped within a RAID consisting of strong RNAPII binding and chromatin looping; whereas the normal liver genes *CXCL2* and *AREG* are only expressed in THLE2 cells and interconnected within a large RAID configuration (Figure 1G). Taken together, our transcriptional analyses (RNA‐seq and RNAPII ChIA‐PET) of using HepG2 cells as a representative of HCC and THLE2 cells as a normal liver control identified and refined a set of HCC‐associated genes that are potential involved in the HCC pathogenesis either uniquely activated (HCC‐specific) or suppressed (HCC‐suppressed) in HCC cells.

### 2.3 Enhancer‐Promoter Interaction Contributes to Regulating Transcription of HCC‐Associated Genes

**Figure 2:**
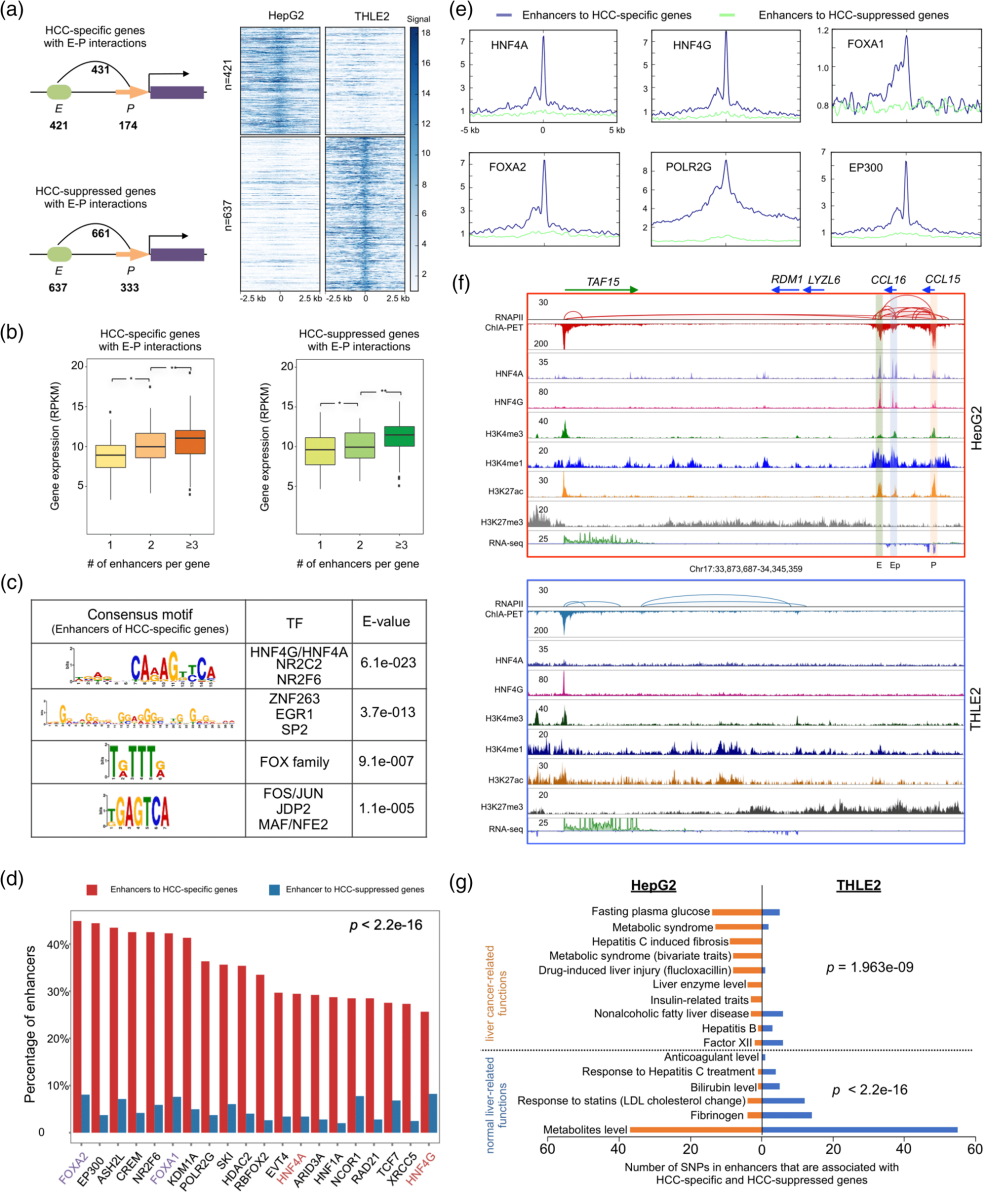
Specific enhancer‐promoter interaction contributes to HCC‐associated gene expression. A) Left panel: schematic representation of HCC‐specific genes (HepG2‐specific) and HCC‐suppressed genes (THLE2‐specific) with associated E‐P interactions in HepG2 and THLE2 cells. Right panel: heatmap showing the H3K4me1 ChIP‐seq enrichment signal centered on the enhancers (± 2.5 kb) involved in E–P interactions. B) Expression level of HCC‐specific and HCC‐suppressed genes with different numbers (1, 2, or > = 3) of connected enhancers in HepG2 and THLE2 cells. *p value < 0.01, **p value < 0.001 via Wilcoxon Rank Sum test. C) Motif scan for enhancers (*n* = 421) connected to HCC‐specific genes via E–P interactions. D) Bar chart of top 20 TFs binding ranked by the percentage of enhancers associated with HCC‐specific genes (red) versus the enhancers associated with HCC‐suppressed genes in in HepG2. ENCODE ChIP‐seq data of 124 TF in HepG2 cells were used for the analysis. (*p *< 2.2e‐16 via two proportion *z*‐test). E) Aggregation plots for liver cancer‐related TFs (FOXA1, FOXA2, HNF4A, and HNF4G) and active transcription‐related TFs (POLR2G and EP300) of ChIP‐seq at enhancers ((±5 kb) associated to HCC‐specific and HCC‐suppressed genes in HepG2 cells. F) Basic browser screenshots (chr17:33873687–34345359) showing RNAPII ChIA‐PET (loops /pkeas) for a multi‐gene (MG) domain involving *CCL16* (weak expression) and *CCL15* (strong expression) with extensive E–P and P–P interactrions. ChIP‐seq tracks of HNF4A/HNF4G and histone marks demarcate enhancer (E), promoter (P), and enhancer‐like promoter (Ep) in HepG2 and THLE2 cells. G) Functions‐related SNPs involved in enhancers that are associated with HCC‐specific genes (left in orange) and HCC‐suppressed genes (right in blue). Liver cancer/impaired liver function‐related and normal liver function‐related SNPs in specific enhancers of HCC‐specific genes (*p* = 1.963e‐09) and HCC‐suppressed genes. Liver cancer‐related functions *p =* 1.963e‐09 and normal liver‐related functions *p *< 2.2e‐16 via hypergeometric test.

Next, we seek to investigate the regulatory mechanism in transcription of HCC‐associated genes. It is known that many protein coding genes are regulated by non‐coding distal *cis*‐acting elements such as enhancers through long‐range chromatin interactions mediated by *trans*‐acting TFs. We first characterized all potential *cis*‐regulatory enhancers that are associated with RNAPII‐mediated chromatin interactions in HepG2 and THLE2 cells, showing that most of the enhancers with RNAPII are in active (H3K4me1+H3K27ac) or intermediate (H3K4me1 only) states, with only a small portion are in poised state (H3K4me1+H3K27me3) (Figure S2A, Supporting Information). Based on our previous study,^[^ ^20^ ^]^ we classified genes that are associated RNAPII‐mediated chromatin interaction domains with either single gene (SG) or multiple genes (MG) (Table S4, Figure S2B,C, Supporting Information). A SG domain possesses enhancer–promoter (E–P) interaction with one promoter, whereas a MG domain includes multiple genes and could have complex chromatin interactions (E–E, E–P, and P–P). Particularly in P–P interactions, not all promoters are equal, and one promoter could be stronger than the other. As the ratio of H3K4me3/3K4me1 ChIP‐seq signals is a quantitative indicator to a given promoter for its strength,^[^ ^20^ ^]^ we calculated the ratio of H3K4me3/H3K4me1 at the TSS (promoter) on HCC‐specific genes in MG domains and assigned the promoter as enhancer‐like (log2 ratio of H3K4me3/H3K4me1 < 0) and strong promoter TSS (log2 ratio of H3K4me3/H3K4me1 >0). In this analysis, we found that 48 (19.6%) out of 245 promoters in MG domains harboring HCC‐specific genes possess enhancer‐like property (Figure S2D,E, Supporting Information) and, thus, likely to enhance the transcription of other HCC‐specific genes. In total, we have identified 421 distal enhancers (including enhancer‐like promoters) that established 431 E–P interactions with 174 out of 386 (45.1%) HCC‐specific genes in HepG2 cells (**Figure** **2**A). We also identified 637 enhancers that were involved in 661 E‐P interactions with 333 out of 924 (36.0%) HCC‐suppressed genes in THLE2 cells (Figure 2A).

To further interrogate the biological function of HCC‐associated genes with E‐P interactions, we performed gene ontology (GO) analysis by Genomic Regions Enrichment of Annotations Tool (GREAT)^[^ ^24^ ^]^ for the 174 HCC‐specific genes interacting with 421 enhancers (Figure 2A). The GO functions of these genes showed association with liver cancer, hepatocellular carcinoma and hyperlipidemia (Figure S2F, Supporting Information), implying that these distal regulatory elements may have important roles. Interestingly, multiple enhancers together exhibited additive efforts in further increasing transcription of HCC‐associated genes via complex E‐P interactions (Figure 2B). This enhancer additive effort, in general, also applies to other genes in HepG2 and THLE2 cells (Figure S2G, Supporting Information).

To explore potential transcription factors that might be participated in specific E‐P interactions with HCC‐associated genes, we performed motif scan analysis using MEME^[^ ^25^ ^]^ on 421 enhancers of HCC‐specific genes (Figure 2A), and identified a number of highly enriched consensus motifs of TF binding (Figure 2C), including the motif for the FOX family (e.g., FOXA1/FOXA2) involved in liver carcinogenesis.^[^ ^26^ ^]^ Among them, the motif for Hepatocyte Nuclear Factor 4 family (HNF4G/HNF4A) is the most significantly enriched. To further validate the specific binding by these TFs in HepG2 at specific enhancers with E‐P interaction, we analyzed ENCODE ChIP‐seq data for 124 TFs in HepG2 cells (Table S5, Supporting Information) for TF binding signals at the genomic coordinates of the corresponding enhancers involved E‐P interaction with HCC‐specific genes and HCC‐suppressed genes. In line with the motif scan analysis, we found that HNF4G/HNF4A and FOXA1/FOXA2 are among the top 20 ranked TFs who's binding signals are significantly enriched in specific enhancers of HCC‐specific genes in HepG2 as in contrast to those enhancers of HCC‐suppressed genes (Figure 2D,E). Other top ranked TFs include EP300 and POLR2G, which are associated with active transcription. As exemplified in Figure 2F, HNF4G/HNF4A binding peaks are specifically strong in *CCL16* enhancer and promoter (enhancer‐like), which interact with the *CCL15* promoter (a strong promoter) as shown by RNAPII ChIA‐PET data, and *CCL15* is actively transcribing as indicated by RNA‐seq data in HepG2 cells. In contrast, there is no HNF4G/HNF4A binding and also no RNAPII present at the *CCL16*/*CCL15* loci, as well as *CCL16*/*CCL15* are inactive in THLE2 cells.

Lastly, we sought to investigate whether non‐coding genetic variants have any impact to regulating HCC‐associated genes. Previous GWAS and linkage studies have identified many gene‐coding variants that are associated with elevated risk for liver cancer.^[^ ^27^ ^]^ However, we have yet to distinguish DNA variants that directly influence risk for liver cancer (i.e., causal variants) from those that are merely in linkage disequilibrium (LD) with them. In particular, it has not been systematically addressed if non‐coding variants in distal regulatory elements may have a role in HCC pathogenesis from a 3D genome perspective. In this direction, we integrated GWAS SNPs with liver/liver disease‐related traits and LD SNPs from the 1000 Genomes Project^[^ ^28^ ^]^ with our E‐P interaction data (Figure S2H, Supporting Information). We found that liver cancer/impaired liver function‐related SNPs were significantly enriched in enhancers of HCC‐specific genes in HepG2 cells as compared to in THLE2 cells, while normal liver function‐related SNPs were more enriched in specific enhancers of HCC‐suppressed genes with E‐P interactions in THLE2 cells (Figure 2G). This result underscores that SNPs may impact enhancer functions and the expression level of genes associated with liver cancer development or normal liver property through modulating the 3D genome structure, specifically enhancer‐promoter interactions.

### 2.4 Co‐Transcription Regulation of HCC‐Associated Genes

**Figure 3:**
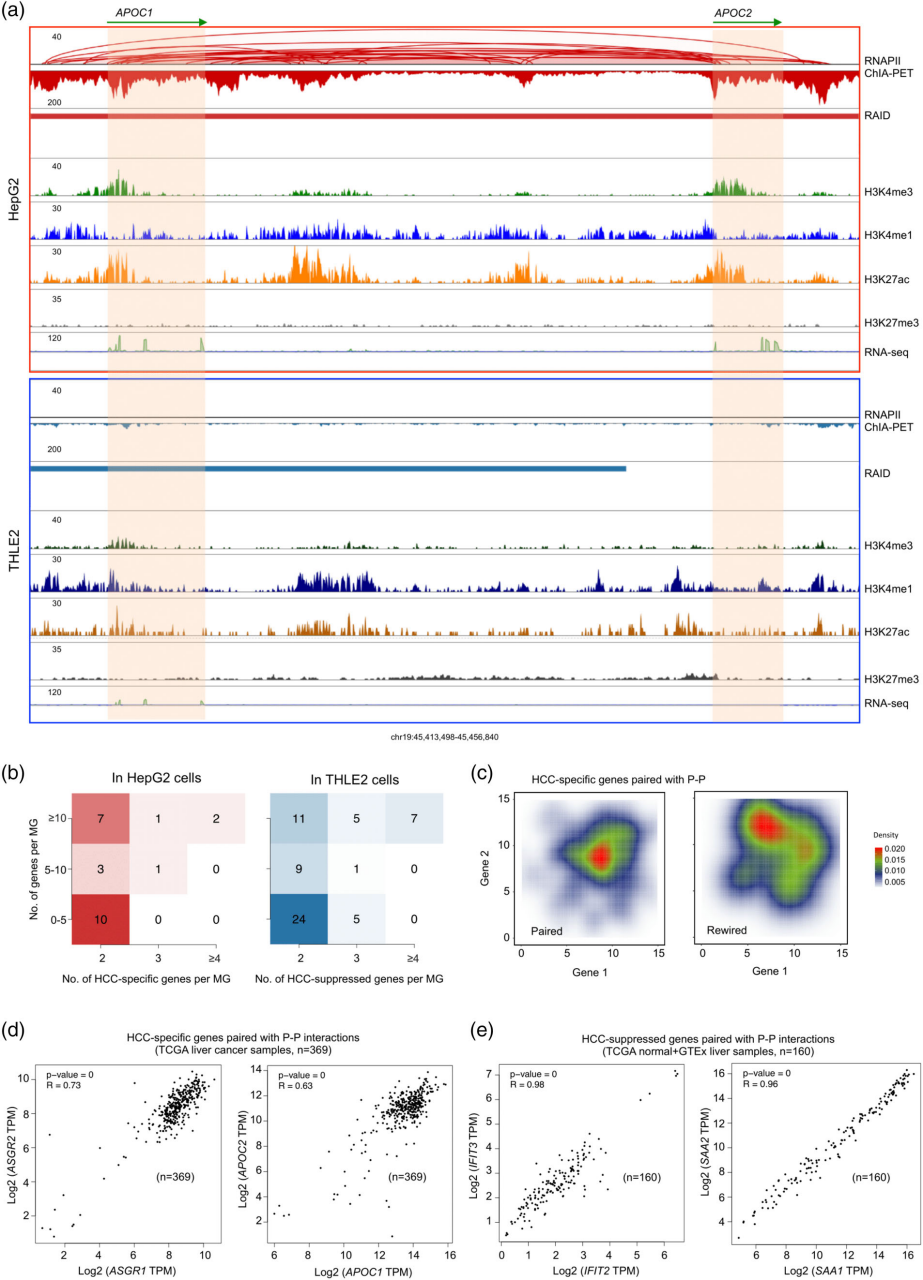
Paired HCC‐associated genes by RNAPII‐associated chromatin interactions are co‐transcription regulated. A) Example view of BASIC browser screenshot (chr19:45413498–45456840) showing a pair of HCC‐specific genes (*APOC1* and *APOC2*) with extensive RNAPII ChIA‐PET and histone ChIP‐seq data revealing substantial E–P and P–P chromatin interactions between enhancers and promoter in HepG2 cells (red box) but not in THLE2 cells (blue box). B) Distribution of HCC‐specific genes (2, 3, ≥4) per multi‐gene complex (MG) in HepG2 (left panel) and HCC‐suppressed genes per multi‐gene complex (MG) in THLE2 (right panel). C) Contour plot of gene expression values (Log2 RPKM) of paired HCC‐specific genes with P–P chromatin interactions in MG (left panel) and randomly rewired gene pairs (right panel). D) Dot‐plots from TCGA data for Liver Hepatocellular Carcinoma (LIHC) (*n* = 369) to illustrate Pearson's correlation for the transcription of HCC‐specific genes (left panel, *ASGR1*; right panel, *APOC1*) with P–P interactions. E) Dot‐plots from TCGA data for TCGA normal and GTEx liver (*n* = 160) to illustrate Pearson's correlation for the transcription of paired HCC‐suppressed genes (left panel, *IFIT2*; right panel, *SAA1*) with P–P interactions.

Intriguingly, we observed that some of the HCC‐specific genes in HepG2 cells and HCC‐suppressed genes in THLE2 cells are located in the same RNAPII‐associated interaction domains with multiple genes (**Figure** **3**A,B, Figure S3A,B, Supporting Information), suggesting a potential topological basis for co‐transcription as we previously observed in other cells.^[^ ^20^ ^]^ Indeed, HCC‐specific genes paired with RNAPII‐associated promoter‐promoter (P–P) interactions showed significant high‐correlation in transcription as measured by RNA‐seq, as in contrast with the randomly rewired the gene pairs (Figure 3C and Figure S3C, Supporting Information). By harnessing TCGA liver cancer RNA‐seq data, we analyzed the expression correlation of HCC‐specific genes in pairs with RNAPII‐associated promoter‐promoter interactions (*n* = 26), and found that 61% (16/26) of gene pairs were transcriptionally correlated in the TCGA data (Pearson's Correlation Coefficient > 0.25) (Figure 3D). Similarly, HCC‐suppressed genes with paired P–P interactions also showed the same trend when analyzed in light of TCGA and GTEx normal liver tissue data (Figure S3D, Supporting Information). For example, *APOC1* and *APOC2* both are HepG2‐specific and are localized in chromosome 19 with 40 000 bp apart. This gene pair (*APOC1* and *APOC2*) are connected by strong RNAPII‐associated chromatin interactions for possible co‐transcription (Figure 3A). However, in THLE2 cells, these two genes are not expressed and lack of RNAPII activities. A pair of HCC‐suppressed genes (*SAA1* and *SAA2*) also showed co‐transcriptional property (Figure S3A, Supporting Information). Taken together, we demonstrate that besides enhancer‐promoter interaction, co‐transcription may provide another mechanism for HCC‐associated gene expression.

### 2.5 CTCF Binding and Looping Proximal to Promoter Regulate Transcription of HCC‐Associated Genes

**Figure 4:**
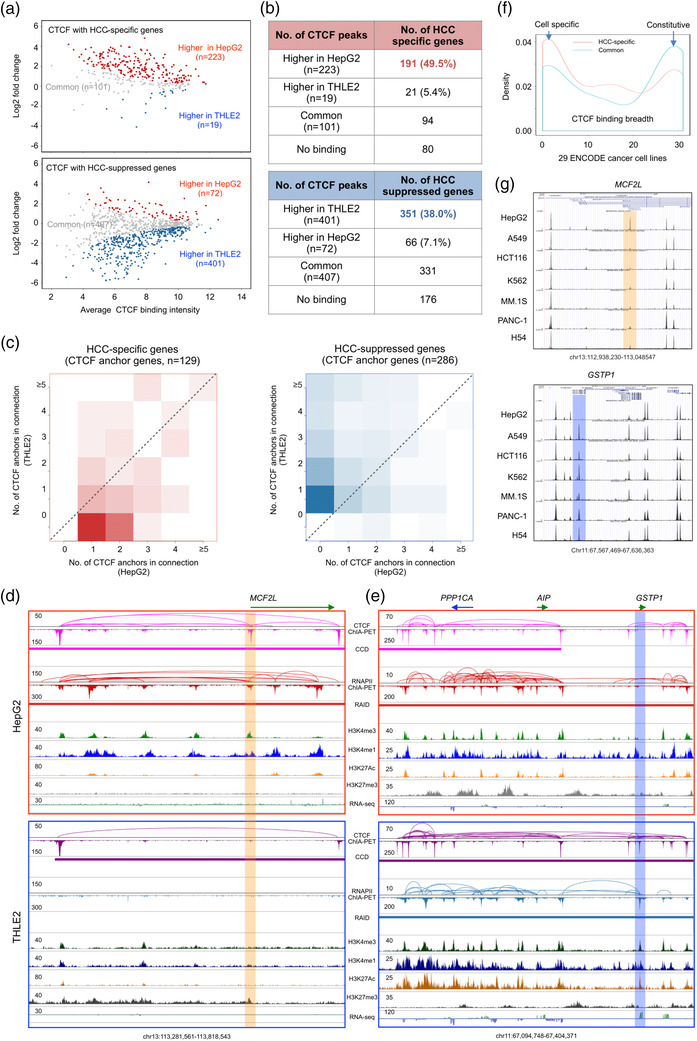
CTCF binding and looping proximal to promoter regulate transcription of HCC‐associated genes. A) MA plots showing CTCF binding peak intensity centered (±2 kb) around TSS of HCC‐specific genes (left panel) and around HCC‐suppressed genes (right panel) in HepG2 and in THLE2 cells. The numbers of CTCF binding sites with signals increased in HepG2 (red), increased in THLE2 (blue), no common (grey) in both cell lines are indicated, respectively. B) Statistics of HCC‐specific genes (left panel) with CTCF binding and HCC‐suppressed genes (right panel) with CTCF binding in HepG2 and THLE2 cells. C) Number of CTCF anchors in chromatin loops in proximal to TSS (promoter) of HCC‐specific genes (left panel) and of HCC‐suppressed genes in HepG2 and THLE2 cells, respectively. D) BASIC browser screenshots showing an example of differential CTCF and RNAPII ChIA‐PET mapping for binding/looping and chromatin interaction domains (CCD and RAID) centered at the TSS of *MCF2L* (a HCC‐specific gene) in HepG2 (top, red box) and in THLE2 (bottom, blue box). Corresponding histone peaks by ChIP‐seq and gene expression by RNA‐sedq are included as epigenomic references. E) BASIC browser screenshots showing an example of differential CTCF and RNAPII ChIA‐PET mapping for binding/looping and chromatin interaction domains (CCD and RAID) centered at the TSS of gene *GSTP1* (a HCC‐suppressed gene) in HepG2 (top, red box) and in THLE2 (bottom, blue box). Corresponding histone peaks by ChIP‐seq and gene expression by RNA‐sedq are included as epigenomic references. F) CTCF binding breadth of CTCF sites with increased CTCF binding intensity and associated with HCC‐specific genes (nonparametric Kolmogorov‐Smirnov test. *p* = 0.04). CTCF binding sites in 29 ENCODE cancer cell lines were used f (https://www.encodeproject.org) were used for analysis. See also Table S6, Supporting Information for the source of CTCF data. G) Browser screenshots for two example views. Top panel shows a strong CTCF binding proximal to the promoter of *MCF2L* (a HCC‐specific gene) in HEPG2 as compared to other 6 different cancer cell lines (A549, HCT116, K562, MM.1S, PANC‐1, H54). This HCC‐specific CTCF binding is highlighted in orange. Low panel shows the lack of CTCF binding proximal to the promoter of *GSTP1* (a HCC‐suppressed gene) in HepG2 as compared to same set of six other cancer cell lines.

To investigate if chromatin folding architectures are changed in HCC cells transformed from normal hepatocytes, we also performed CTCF ChIA‐PET experiments and characterized higher‐order chromatin compartment, chromatin domains, and chromatin loops mediated by CTCF and RNAPII in HepG2 and THLE2 cells using the ChIA‐PET datasets (Table S1, Supporting Information). At compartment and domain levels, the two 3D genomes were largely conserved. (Figure S4A,B, Supporting Information). The estimated compartment switch from B (in THLE2)→A (in HepG2) affected <15% of HCC‐specific genes (Figure S4C, Supporting Information). For example, *STXBP6* is expressed in HepG2 but not in THLE2 cells, where this gene in THLE2 is located in a B compartment (inactive); whereas in HepG2 it is in an A compartment and associated with extensive histone marks of active transcription as well as RNAPII mediated chromatin loops (Figure S4D, Supporting Information). Nonetheless, we identified large numbers of CTCF binding sites that exhibited significant differential binding intensity between HepG2 and THLE2 cells (Figure S4E, Supporting Information). Specifically, 223 CTCF binding sites proximal to the promoter of 191 (49.5%) HCC‐specific genes showed higher binding intensity in HepG2 and 401 CTCF binding sites proximal to the promoter of 351 (38.0%) HCC‐suppressed genes had higher CTCF binding intensity in THLE2 cells (**Figure** **4**A,B). Furthermore, we observed that 129 (33.4%) HCC‐specific genes in HepG2 and 286 (31.0%) HCC‐suppressed genes in THLE2 showed cell‐specific CTCF looping involving the gene promoters (Figure 4C), implying a correlation between CTCF looping on the promoter and differential gene expression. This is in line with our previous report showing that genes proximal to the anchors of CTCF loops (CTCF anchor genes) were associated with active transcription.^[^ ^14^ ^]^ For instance, *MCF2L*, an HCC‐specific gene (Figure S4F, Supporting Information), had specific CTCF binding/looping proximal to its promoter and is associated with considerable RNAPII binding/looping (Figure 4D) in HepG2 but absent in THLE2 cells. On the contrary, *GSTP1*, known as a tumor suppressor in HCC,^[^ ^29^ ^]^ is highly expressed in THLE2 and showed a strong CTCF binding peak proximal to *GSTP1*’s promoter along with extended CTCF and RNAPII mediated chromatin loops, but completely off in HepG2 (Figure 4E), implying the loss of this specific CTCF binding and associated chromatin loops that lead to a possible displacement of RNAPII during transformation from normal hepatocyte to HCC. To further validate the specificity of CTCF binding sites proximal to HCC‐associated genes, we integrated the CTCF ChIP‐seq data from ENCODE (Table S6, Supporting Information) and ascertained that these CTCF binding sites are indeed enriched as cell‐specific when compared to 29 ENCODE cancer cell lines (Figure 4F) and 70 non‐cancer cell lines (Figure S4G, Supporting Information). As exemplified from the browser screenshot including 7 ENCODE cell lines derived from different type of cancers, the presence of the CTCF binding proximal to *MCF2L* promoter in HepG2 as compared to other six cancer cell lines is likely specific gain of function to HCC instead of some general cancer property, while the absence of the CTCF binding next to *GSTP1* promoter in HepG2 may indicate a possible loss of function in HCC (Figure 4G). Collectively, our observations suggest that specific CTCF binding and looping proximal to promoter is associated with HCC‐associated transcription regulation.

### 2.6 Altered CTCF Binding is Linked to CpG Methylation in CTCF Binding Motif

**Figure 5:**
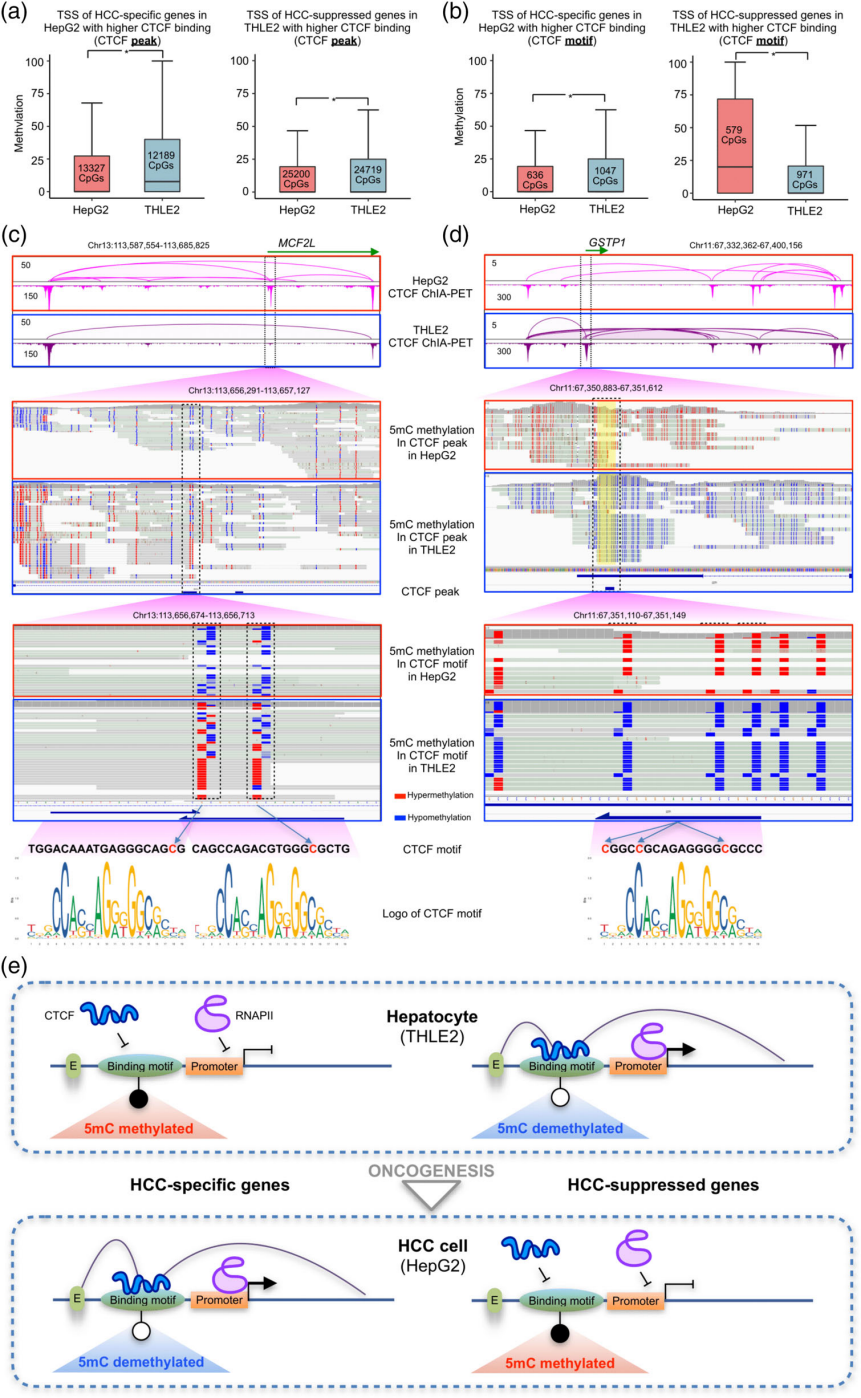
Altered CTCF binding is linked to CpG methylation in CTCF binding motif. A) Box plots showing methylation level in both HepG2 and THLE2 cells of CTCF peak regions proximal (±2 kb) to TSS of HCC‐specific genes with higher CTCF binding in HepG2 cell (left panel) and to the HCC‐suppressed genes with higher CTCF binding in THLE2 cell (right panel). *p value < 0.01 via Wilcoxon Rank Sum test. B) Box plots showing methylation level in both HepG2 and THLE2 cells of CTCF motif in the CTCF peaks proximal (±2 kb) to TSS of HCC‐specific genes with higher CTCF binding in HepG2 (left panel) and of HCC‐suppressed genes with higher CTCF binding in THLE2 (right panel. *p value < 0.01 via Wilcoxon Rank Sum test. C) Browser screenshots showing an example of methylation in CTCF binding region proximal to the TSS of *MCF2L* at levels of CTCF binding peak and CTCF motif in both HepG2 and THLE2 cells. The observed hypermethylation (red) and hypomethylation (blue) are highlighted. Differential methylated cytosine (DMC) is highlighted in yellow. D) Browser screenshots showing an example of methylation in CTCF binding region proximal to the TSS of *GSTP1* at the levels of CTCF peak and CTCF motif. E) Schematic model ellucidating potential alterations of methylation on CTCF binding motif that could modulate CTCF binding affinity and chromatin loop formation, and lead to altered transcription regulation of HCC‐specific and HCC‐suppressed genes.

In order to interrogate the underlying mechanism of differential CTCF binding in HCC and normal hepatocyte cells, we first examined the genetic variation in the CTCF binding regions. We integrated the liver and liver disease‐related SNPs from GWAS and WGS data of HepG2 and THLE2 (Table S1, Supporting Information) but did not find significant contribution of non‐coding SNPs or small indels in the loci of CTCF binding sites or motifs near the TSSs of HCC‐associated genes (Figure S5A, Supporting Information), which motivated us to examine for potential epigenetic clue of differential CTCF binding. As DNA methylation is linked to transcription factor occupancy^[^ ^30^ ^]^ and CTCF in genomic imprinting,^[^ ^31^ ^]^ we performed whole genome bisulfite sequencing (WGBS) in HepG2 and THLE2 cells (Table S1, Supporting Information) for extensive genome‐wide analysis. Approximately 50 million CpG sites for each of the two cell lines were identified, which is comparable to the HepG2 WGBS data previously analyzed in the ENCODE project (Figure S5B, Supporting Information). Moreover, clustering analysis indicated that the HepG2 WGBS data generated in this study are highly correlated with those generated from ENCODE (Figure S5C, Supporting Information), suggesting high quality DNA methylation data for downstream analysis. We found that the HepG2‐ and THLE2‐specific CTCF binding peak sites (≈500 bp) and CTCF binding motifs (219 bp) proximal to the TSS of HCC‐specific genes in HepG2 and HCC‐suppressed genes in THLE2 are both hypomethylated, respectively (**Figure** **5**A,B), which is in line with the overall CTCF binding peaks in HepG2 and THLE2 cells, respectively (Figure S5D,E, Supporting Information). Furthermore, we identified consistent changes in the hypomethylation of CTCF motifs in HepG2‐ and THLE2‐specific CTCF binding peaks. For example, at the promoter locus (TSS) of *MCF2L*, the zoom‐in browser screenshots of bisulfite DNA methylation data in CTCF binding motifs (Figure 5C) showed high level of hypomethylation in HepG2 cells and hypermethylation in THLE2 cells. On the contrary, at the promoter site of *GSTP1*, the bisulfite DNA methylation data in CTCF binding motif showed a complete opposite view, high level of hypermethylation in HepG2 cells and hypomethylation in THLE2 cells (Figure 5D). Taken together, our observations suggest that altered CTCF binding proximal to promoter is linked to CpG methylation on the CTCF binding sites (motif), leading to differential chromatin looping and transcription of HCC‐associated genes in the transformation from normal hepatocytes to HCC cells (Figure 5E).

## 3 Discussion

In this study, we leveraged multi‐omics analysis to shed light on the 3D epigenomic features and their impacts on transcriptional regulation in HCC cells. First, we identified a significant number of HCC‐associated genes (HCC‐specific and HCC‐suppressed), whose promoters and distal enhancers were in connection in long‐range distance in forms of interaction clusters often involving multiple genes and regulatory elements, in line with previous reports.^[^ ^12^, ^20^, ^22^, ^32^ ^]^ This mechanism may, at least in part, explain why some of the HCC‐associated genes are co‐transcribed together. Recently, STARR‐seq^[^ ^33^, ^34^ ^]^ and CREST‐seq^[^ ^35^ ^]^ have been established to experimentally interrogate the functionality of *cis*‐regulatory elements at genome‐wide scale, which further supported that spatial contacts detected by ChIA‐PET and Hi‐C possess functional implementation. To this end, further study will be informative to characterize the identity of promoters with potential enhancer activity in HCC cells and to test the consequence of the co‐expression model by perturbation. Moreover, we observed that the number of connected enhancers (typical enhancer and enhancer‐like promoter) is significantly associated with gene expression level, suggesting that enhancer activity quantitatively contributes to transcription.

As TFs are key determinants of enhancer activity, we hypothesized that the TFs enriched in HepG2‐specific and connected enhancers would be implicated in HCC transformation. As a proof of principle, we performed TF motif scan on enhancers with E–P interactions to HCC‐associated genes and identified HNF4G/HNF4A as the most enriched TF motif. Roles for these two transcription factors in HCC have not been previously described, but supported by a recent study showing that HNF4A is upregulated in livers of nonalcoholic steatohepatitis (NASH) patients and positively correlated with nonalcoholic fatty liver disease (NAFLD) fibrosis score,^[^ ^36^ ^]^ together suggesting HNF4A a central TF in the pathogenesis of NASH. Although the potential function of HNF4G in HCC is unknown, a recent study revealed that HNF4G is a pioneer factor that reprograms the enhancer landscape at gastrointestinal genes and mediates androgen‐receptor therapy resistance in prostate cancer.^[^ ^37^ ^]^ We thus propose that HNF4G/HNF4A may activate the expression of NASH‐associated genes via E‐P interaction, potentially leading to liver fibrosis/cirrhosis and HCC.

In addition, we found that the majority of RNAPII‐associated interaction domains (RAIDs) are within, or overlapping with, CTCF‐mediated chromatin chromatin domains (CCDs), suggesting that CTCF‐mediated chromatin architecture may provide the topological framework that may constrain transcription regulation. We also found that the overall CCD distribution is conserved between HepG2 and THLE2 cells. However, unexpectedly, we discovered that the majority of HCC‐specific genes in HepG2 (HCC) cells have concomitant increase of CTCF binding comparing to THLE2 cells, whereas the HCC‐suppressed genes showed higher CTCF binding and looping in THLE2 cells than in HepG2 cells. To gain mechanistic insight into the differential CTCF binding on the promoter, we first examined the DNA sequence in CTCF binding sites for potential course of genetic variants, but found only a tiny fraction of DNA sequence variation. Remarkably, our analysis of whole genome bisulfite DNA sequencing revealed that the differential CTCF binding and extended looping associated with HCC‐specific genes in normal hepatocytes and HCC cells are highly correlated with epigenetyic states of 5mC methylation or demethylation, suggesting a robust imprinting and reprogramming mechanism involved in regulating the dynamics of DNA methylation/demethylation that in turn alter chromatin folding architecture and control transcription of HCC‐associated genes during the transformation from normal hepatocytes to HCC cells. Recently developed tools for epigenome editing provided evidence that the DNA methylation status can be modulated by dCas9/TALE fused with engineered DNA (de)methylation (e.g., DNMT and TET)^[^ ^38^, ^39^ ^]^ and maintained in vivo.^[^ ^40^ ^]^ A recent report described an improved dCas9 method to precisely modulate mCG states at TF binding motifs at single base resolution to clarify the roles of mCG in shaping TF occupancy.^[^ ^41^ ^]^ Future investigations in applying those tools will be valuable to test whether perturbation of DNA methylation on the CTCF motif in the promoter of HCC‐specific genes in HepG2 and HCC‐suppressed genes in THLE2 can reprogram the 3D genome architecture and subsequent enhancer–promoter interaction and transcription via reconfiguring CTCF binding and loop formation. Collectively, as DNA hypomethylation is a known hallmark of HCC,^[^ ^42^, ^43^ ^]^ methylation‐dependent changes to CTCF binding may represent a previously unrecognized mechanism during HCC pathogenesis through its genome‐wide impact on 3D chromatin structure. Thus, modulation of CTCF binding by therapeutic epigenome editing may evolve as a novel strategy for future HCC treatment.

## 4 Experimental Section

### Cell Culture

HepG2 (ATCC HB‐8065) and THLE2 (ATCC CRL‐2706) cell lines were purchased from ATCC. HepG2 cells were cultured in DMEM supplemented with 10% FBS and 100 U mL^−1^ penicillin/streptomycin. THLE2 cells were cultured by using BEGM Bullet Kit (Lonza/Clonetics Corporation, cat.#CC3170). The culture dish used for THLE2 were precoated with a mixture of 0.01 mg mL^−1^ fibronectin, 0.03 mg mL^−1^ bovine collagen type I and 0.01 mg mL^−1^ bovine serum albumin dissolved in BEBM medium.

### Long‐Reads ChIA‐PET

Long Reads ChIA‐PET libraries with antibody against RNAPII and CTCF were generated with HepG2 and THLE2 cells by following the ChIA‐PET protocol reported previously.^[^ ^14^, ^44^ ^]^ Briefly, when the cells were grown to 80% confluency, 40 mL of ethylene glycol bis (EGS) (Thermo Fisher Scientific, cat.#21565)/1 X PBS solution was added to each culture dish followed by shaking on orbital shaker for 45 min at room temperature (RT). After that, 1.1 mL of 37% formaldehyde (final concentration: 1%) (EMD Millipore, cat. # 344198) was added to each dish followed by shaking on orbital shaker for 20 min at RT. Subsequently, 3.57 mL of 2.5 m glycine (final concentrate: 0.2 m) (Sigma‐Aldrich, cat# G8898) was added to each dish followed by shaking on orbital shaker for 10 min at RT. The cells were scraped off the dish by cell scraper and transferred into 50 mL corning tube and then centrifuged at 2000 rpm for 10min, 4 °C. Media was discarded by pipetting and the cells were washed for twice by adding 20 mL chilled PBS and then centrifuged at 2000 rpm for 5 min, 4 °C. After removing the supernatant, the cells pellets were stored at −80 °C.

300 millions of fixed HepG2/THLE2 cells were used to generate one ChIA‐PET library. Briefly, after cell lysis and nuclear lysis, the nucleus was sonicated into ≈1 kb size. Then chromatin was precleared using protein G beads and immunoprecipitation was performed using anti‐RNAPII (BioLegend, cat.# 8WG16)/anti‐CTCF antibody (Abcam, cat.# ab70303) coated protein G beads (Life Technologies, cat. no. 10009D). Then on‐beads A‐tailing was performed using Klenow Fragment (3′>5′ exo‐) (NEB, cat.#M0212L) and dATP (100 mm; NEB, cat.#N0440S). On‐bead proximity ligation was performed using in‐house bridge linker (F: 5′‐ /5Phos/CGCGATATC/iBIOdT/TATCTGACT ‐3′, R: 5′‐ /5Phos/GTCAGATAAGATATCGCGT ‐3′. HPLC purified, from Integrated DNA Technologies) and T4 DNA ligase (Thermo Fisher Scientific, cat.#EL0013). After that, proximity ligated chromatin complexes were eluted from beads and proteinase K (Life Technologies, cat.#AM2548) was added to reverse cross‐linked chromatin complexes for overnight. After Phenol:chloroform:IAA (Ambion, cat.#AM9730) extraction and isopropanol precipitation, tagmentation was performed on the proximity‐ligated DNA by Tn5 transposase from Nextera DNA Sample Prep Kit (Illumina, cat.#FC‐121‐1031). Then tagmented DNA was immobilized on M280 streptavidin dynabeads (Invitrogen, cat.#11205D). PCR amplification was performed on beads using Nextera DNA Sample Prep Kit and the products were purified by AMPure XP beads (Beckman, cat.#A63881) and subjected to size selection (300–600 bp) on BluePippin instrument (Sage Science) using Blue Pippin Cassette Kit (Sage Science, cat.#BDF2010). DNA library was sequenced on Illumina Nextseq 500 by paired‐end 150 bp.

### ChIP‐seq

Ten million of HepG2 and THLE2 cells were fixed by adding 1% (final concentration) formaldehyde (EMD Millipore, cat# 344198). Fixation was stopped by the addition of 0.125 m glycine (final concentration) (Sigma‐Aldrich, cat# G8898). Chromatin was isolated by the addition of lysis buffer and lysates were sonicated and the DNA sheared to an average length of 200–300 bp by Bioruptor (Diagenode). Genomic DNA (Input) was prepared by treating aliquots of chromatin with proteinase K (Life Technologies, cat.#AM2548) at 65 °C for de‐crosslinking overnight, followed by purification using MinElute PCR Purification Kit (Qiagen, cat.# 28004). Sonicated chromatin was precipitated by H3K4me1 (Abcam, cat.#ab8895), H3K4me3 (Abcam, cat.#ab8580), H3K27Ac (Abcam, cat.#ab4729), H3K27me3 (Millipore, cat.#07‐449) antibodies. Precipitated chromatin complexes were washed, eluted from the beads with SDS buffer, and subjected to proteinase K treatment. De‐crosslinking was performed by incubation overnight at 65 °C. ChIPed DNA was purified by Qiagen MinElute kit. Library preparation was performed using the KAPA Hyper Prep kit (Roche, cat.#07962363001) and sequenced on Illumina Nextseq 500 by single‐end 50 bp.

### RNA‐seq

Total RNA was extracted from HepG2 and THLE2 using PureLink RNA Mini kit (Thermo Scientific, cat.#12183020) and subjected to on‐column DNase I (Thermo Scientific, cat.#18068015) treatment. 1 µg RNA was performed ribosomal RNA depletion using the riboZero rRNA removal kit (Illumina, cat.# MRZH11124). RNA was fragmented into 200 bp in 5X NEBNext First Strand Synthesis Reaction Buffer (New England BioLabs, cat.#E7525S). First strand cDNA was synthesized using random hexamer primer and M‐MuLV Reverse Transcriptase (New England BioLabs, cat.#M0253S). Second strand cDNA synthesis was subsequently performed using DNA Polymerase I (New England BioLabs, cat.# M0209S) and RNase H (New England BioLabs, cat.# M0297S). Library preparation was performed using the KAPA Hyper Prep kit (Roche, cat.#07962363001). DNA library was sequenced on Hiseq 2500 by paired‐ end 100 bp.

### Whole Genome Sequencing

A total amount of 2 µg DNA per sample (HepG2/THLE2) was used as input material for the DNA library preparations. Sequencing library was generated using Truseq Nano DNA HT Sample Prep Kit (Illumina) as per manufacturer's instruction and index codes were added to each sample. Briefly, genomic DNA sample was fragmented by sonication to a size of 300–500 bp by Covaris M220. Then DNA fragments were end‐polished, A‐tailed, and ligated with the full‐length adapter for Illumina sequencing, followed by further PCR amplification. After PCR products were purified using AMPure beads, libraries were analyzed for size distribution by Agilent 2100 Bioanalyzer and quantified using KAPA Hyper Prep kit (Roche, cat.#07962363001) and sequenced on Illumina HiSeqXten by pair‐end 150 bp.

### Whole Genome Bisulfate Sequencing

2 µg of HepG2/THLE2 genomic DNA was sonicated using a Covaris M220 into a size of 300–500 bp. Sodium bisulfite conversion of all DNA samples was performed using the EZ DNA Methylation kit (Zymo EZ DNA Methylation Kit, Zymo Research). All libraries were subjected to quality control by Agilent Bioanalyzer examination and quantified using the KAPA Hyper Prep kit (Roche, cat.#07962363001) and sequenced on Illumina HiSeqXten by pair‐end 150 bp.

### Methods for Quantification and Statistical Analysis

See Supporting Information.

### Availability of Data and Materials

Genome‐wide sequencing reads were deposited at GEO. The accession number for the ChIA‐PET, ChIP‐Seq, whole genome sequencing, and RNA‐Seq datasets for HepG2 and THLE2 cells reported in this paper is GEO: GSE144893. Software used in this study is listed in the Software and Algorithms Table.

## Conflict of Interest

The authors declare no conflict of interest.

## Author Contributions

Y.F., P.W., L.C., and M.Z. contributed equally to this work. X.C., and L.L. conceptualized and supervised this study. Y.F., P.W., and M.Z. designed the experiments and generated all genomic data. L.C. analyzed the data. E.G. cultured cells and treated cells. J.W. and S.L. processed the whole genome bisulfate sequencing data and provided valuable suggestions. F.H., Y.L., W.Z., W.Z., Y.X., X.H. provided expertise in liver cancer biology and contributed to manuscript preparation. Y.F., P.W, L.C., and Y.R. interpreted the results and wrote the manuscript.

## Supporting information

Supporting Information

## Data Availability

The data that support the findings of this study are available in the supplementary material of this article.
