# Supplementary material for: 3D‐Epigenomic Regulation of Gene Transcription in Hepatocellular Carcinoma
Source: Adv Genet (Hoboken). 2022 Jun 29;3(4):2100010. doi: 10.1002/ggn2.202100010 (PMC9993472; doi:10.1002/ggn2.202100010)
Supplement: Supplementary file 1 — Supporting Information [file GGN2-3-2100010-s001.pdf]

ADVANCED GENETICS
Open Access

Supporting Information

for *Advanced Genetics*, DOI 10.1002/ggn2.202100010

3D-Epigenomic Regulation of Gene Transcription in Hepatocellular Carcinoma

Yuliang Feng, Ping Wang, Liuyang Cai, Meixiao Zhan, Fan He, Jiahui Wang, Yong Li, Eva Gega, Wei Zhang, Wei Zhao, Yongjie Xin, Xudong Chen, Yijun Ruan and Ligong Lu**

ADVANCED GENETICS
Open Access

Supporting Information

for *Advanced Genetics*, DOI 10.1002/ggn2.202100010

3D-Epigenomic Regulation of Gene Transcription in Hepatocellular Carcinoma

Yuliang Feng, Ping Wang, Liuyang Cai, Meixiao Zhan, Fan He, Jiahui Wang, Yong Li, Eva Gega, Wei Zhang, Wei Zhao, Yongjie Xin, Xudong Chen, Yijun Ruan and Ligong Lu**

SUPPLEMENTAL INFORMATION

3D-Epigenomic Regulation of Gene Transcription in Hepatocellular Carcinoma

Yuliang Feng[1,2,4], Ping Wang[2,4], Liuyang Cai[2,4], Meixiao Zhan[1,4], Fan He[3], Jiahui Wang[2], Yong Li, Eva Gega[2], Wei Zhang[3], Wei Zhao[1], Yongjie Xin[1], Xudong Chen[3*], Yijun Ruan[2*], Ligong Lu[1*]

SUPPLEMENTAL FIGURES (Figure S1-S5) ……………………………………………………………P2

SUPPLEMENTAL TABLES (Table 1-6) ……………………………………………………………P10

SUPPLEMENTAL METHODS ……………………………………………………………………P19

SUPPLEMENTAL REFERENCES…………………………………………………………………P27

SUPPLEMENTAL FIGURES

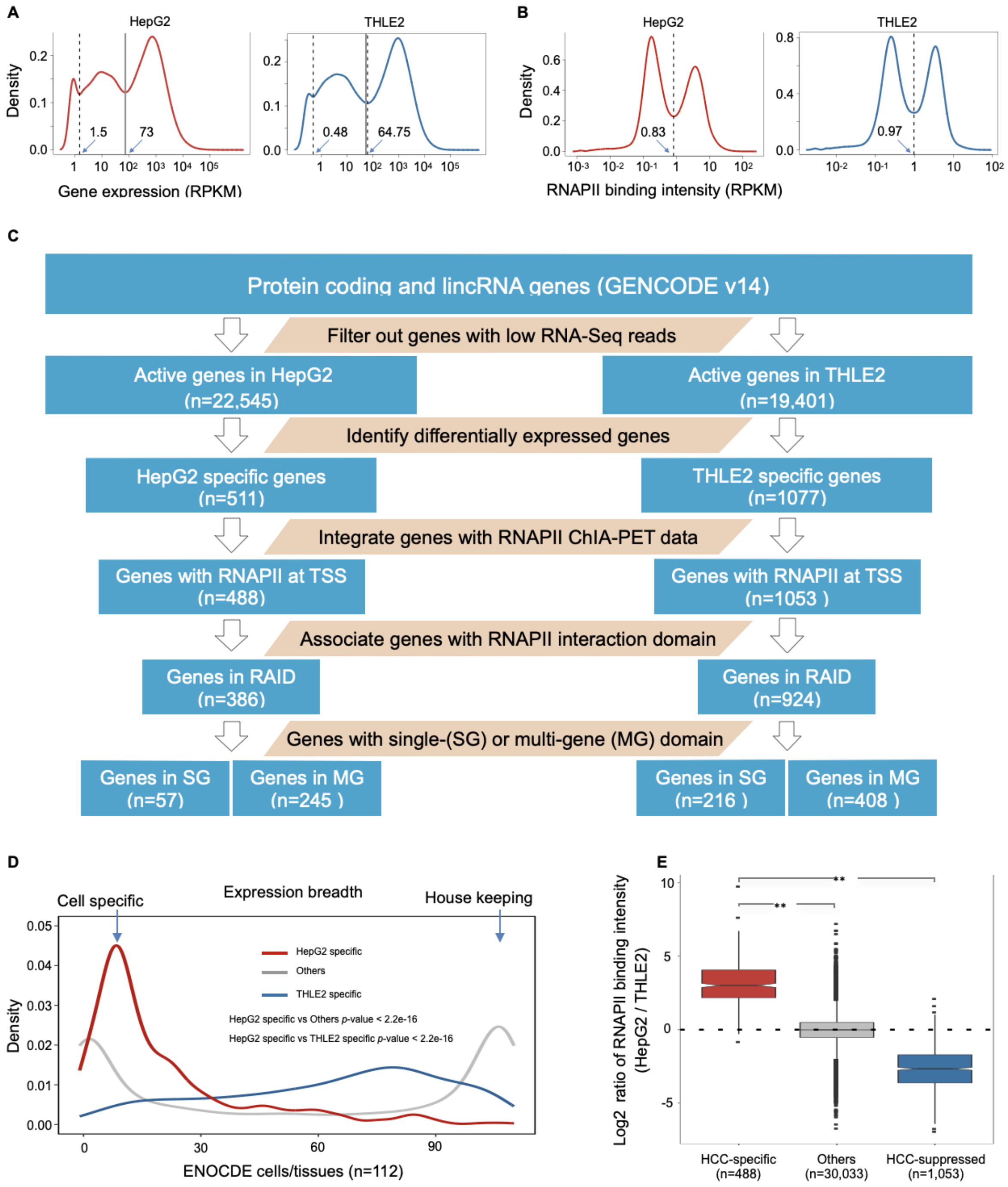

Figure S1. **Identification of HCC-specific genes using RNA-seq and ChIA-PET data.** Related to Figure 1.

A-B. Kernel density plot distributions of normalized RNA-seq counts (RPKM) in (A) and normalized RNAPII binding intensity (RPKM) in (B) in HepG2 and THLE2 cells, respectively. The genes with low expression (RPKM <1.5 in HepG2 and <0.48 in THLE2) and low RNAPII binding on their promoters (RPKM <0.83 in HepG2 and <0.97 in THLE2) were removed from

downstream analysis. **C**. Flow chart of filtering steps using gene expression (RNA-seq) and chromatin interaction (RNAPII ChIA-PET) data for the identification of differentially expressed genes in HepG2 and THLE2 cells and further characterization for genes that are associated within RAID (RNAPII associated interaction domain) and MG (multi-gene complex). **D**. Gene expression breadth (number of different tissues a gene is expressed in) of HepG2 and THLE2 specific genes in 112 tissues/cells from ENCODE data. HepG2 specific genes (red) are significantly represented as tissue-specific (expressed in only a few cells/tissues, while the THLE2 specific genes (blue) are mostly constitutive (expressed in many cells/tissues). The grey curve is for all other genes as a reference (nonparametric Kolmogorov-Smirnov test. p< 2.2e-16). **E.** Box plot showing the ratio (HepG2/THLE2) of RNAPII binding density in HepG2 and THLE2 at promoters of HCC-specific genes (red) and HCC-suppressed genes (blue). The The non-differential genes (grey) are included as a reference.

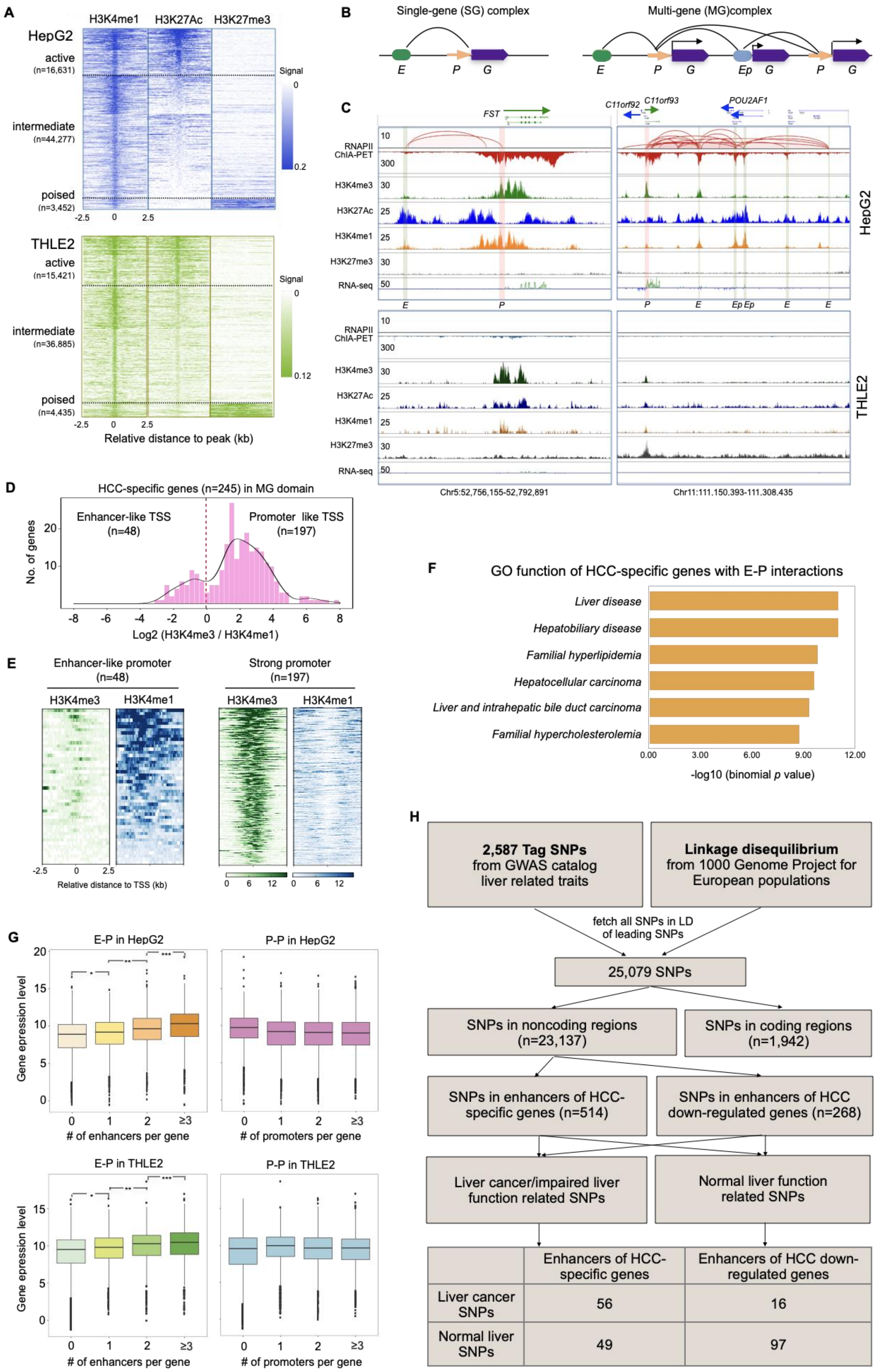
A
H3K4me1
H3K27Ac
H3K27me3
HepG2
active
(n=16,631)
intermediate
(n=44,277)
poised
(n=3,452)
Signal
0
0.2
-2.5
0
2.5
THLE2
active
(n=15,421)
intermediate
(n=36,885)
poised
(n=4,435)
Signal
0
0.12
Relative distance to peak (kb)
B
Single-gene (SG) complex
Multi-gene (MG)complex
E
P
G
Ep
C
FST
C11orf92
C11orf93
POU2AF1
RNAPII ChIA-PET
H3K4me3
H3K27Ac
H3K4me1
H3K27me3
RNA-seq
10
300
30
25
50
HepG2
THLE2
Chr5:52,756,155-52,792,891
Chr11:111,150,393-111,308,435
D
HCC-specific genes (n=245) in MG domain
Enhancer-like TSS
(n=48)
Promoter like TSS
(n=197)
No. of genes
Log2 (H3K4me3 / H3K4me1)
E
Enhancer-like promoter
(n=48)
Strong promoter
(n=197)
H3K4me3
H3K4me1
Relative distance to TSS (kb)
F
GO function of HCC-specific genes with E-P interactions
Liver disease
Hepatobiliary disease
Familial hyperlipidemia
Hepatocellular carcinoma
Liver and intrahepatic bile duct carcinoma
Familial hypercholesterolemia
-log10 (binomial p value)
G
E-P in HepG2
P-P in HepG2
E-P in THLE2
P-P in THLE2
Gene epression level
# of enhancers per gene
# of promoters per gene
H
2,587 Tag SNPs
from GWAS catalog
liver related traits
Linkage disequilibrium
from 1000 Genome Project for
European populations
fetch all SNPs in LD
of leading SNPs
25,079 SNPs
SNPs in noncoding regions
(n=23,137)
SNPs in coding regions
(n=1,942)
SNPs in enhancers of HCC-specific genes (n=514)
SNPs in enhancers of HCC down-regulated genes (n=268)
Liver cancer/impaired liver function related SNPs
Normal liver function related SNPs
Enhancers of HCC-specific genes
Enhancers of HCC down-regulated genes
Liver cancer SNPs
56
16
Normal liver SNPs
49
97

Figure S2. Enhancer-promoter interaction contributes to HCC-specific gene expression. Related to Figure 2**.**

A. Heatmaps of histone mark ChIP-seq data showing the distribution of all enhancers in three categories (1. active: H3K4me1+H3K27Ac; 2. intermediate: H3K4me1 only; 3. poised: H3K4me1+H3K27me3) in HepG2 (top panel) and THLE2 (bottom panel). Normalized density of H3K4me1 and H3K27ac enrichment is centered on the binding peak summit in ± 2.5 kb window. Color key represents the signal density, where darker blue or green represents higher ChIP-seq signal. **B**. Model cartoon of single-gene (SG) complex interaction and multi-gene (MG) complex interaction with functional features indicated: enhancer (*E*), promoter (*P*), enhancer-like promoter (*Ep*), and gene (*G*). **C**. Example views of browser screenshots showing single-gene (SG) of *FSF* and multi-gene (MG) (*C11orf92-C11orf93-POU2AF1 loci*) complex interactions harboring HCC-specific genes (HepG2-specific) with tracks of RNAPII ChIA-PET (loops/peaks), histone ChIP-seq, and RNA-seq in HepG2 and THLE2 cells. Enhancer (*E*), promoter (*P*), and enhancer-like promoter (*Ep*) are highlighted in green and red, respectively. **D**. Distribution of HCC-specific genes in HepG2 MG model based on enrichment signal ratio (H3K4me3/H3K4me1) centered on TSS in a window of ± 2.5 kb. The distribution curve indicates two population of TSS, the enhancer-like and promoter-like TSS. **E**. Heatmaps of histone ChIP-seq data showing the H3K4me3 and H3K4me1 binding signal centered on enhancer-like TSS (n=48) and promoter-like TSS (n=197) for HCC-specific genes in HepG2 MG in a window of ±2.5 kb. **F**. GREAT analysis for specific enhancers with E-P interactions connected to HCC-specific genes. **G**. Expression level of all genes involved with long-range chromatin interactions. Left: different numbers (0, 1, 2 or ≥3) of enhancers connected to a target gene promoter (E-P) in HepG2 and THLE2 (left low panel). Right: different numbers (0, 1, 2 or ≥3) of promoters connected to a target gene promoter (P-P) in HepG2 (right up panel) and THLE2 (right low panel). **p* value<0.01, ***p* value<0.001, ****p* value<0.0001 via Wilcoxon Rank Sum Test. **H**. Flow chart of data processing pipeline for the identification of regulatory GWASs SNPs in HepG2- and THLE2-specific enhancers.

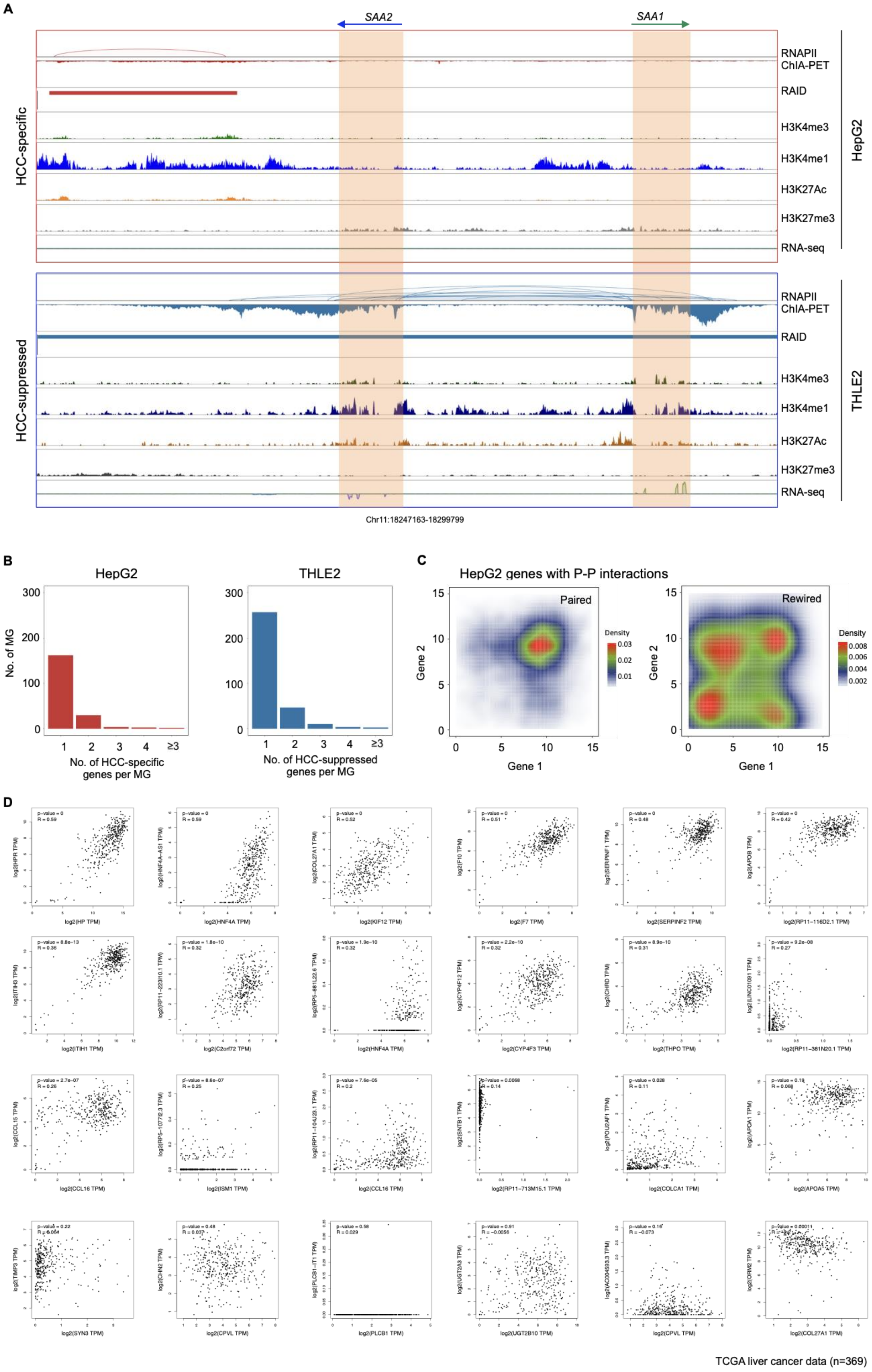
A
SAA2
SAA1
HCC-specific
HCC-suppressed
RNAPII ChIA-PET
RAID
H3K4me3
H3K4me1
H3K27Ac
H3K27me3
RNA-seq
HepG2
THLE2
Chr11:18247163-18299799
B
HepG2
THLE2
No. of MG
No. of HCC-specific genes per MG
No. of HCC-suppressed genes per MG
C
HepG2 genes with P-P interactions
Paired
Rewired
Density
Gene 1
Gene 2
D
TCGA liver cancer data (n=369)

Figure S3. Paired HCC-specific genes by RNAPII-associated chromatin interactions are co-transcription regulated. Related to Figure 3.

A. An example of BASIC browser screenshot showing a pair of HCC-suppressed genes (*SAA1*, *SAA2*) with extensive RNAPII ChIA-PET and histone ChIP-seq data revealing abundant E-P and P-P chromatin interactions between these two genes in THLE2 but not in HepG2 cell. **B**. Distribution of numbers of HCC-specific genes (HepG2-specific) per MG in each RAID in HepG2 cell (left panel) and the numbers of HCC-suppressed genes (THLE2-specific) per MG in each RAID in THLE2 cell (right panel). **C**. Contour plot of gene expression values (Log2 RPKM) for all P-P gene pairs in MG (left panel) and randomly rewired gene pairs (right panel) from HepG2 RNAPII chromatin interaction data. **D**. Scattered dot-plots from TCGA data for Liver Hepatocellular Carcinoma (LIHC) (n=369) to illustrate Pearson's correlation for the transcription of 24 HepG2-specific genes with P-P interactions (*HP* and *HPR*, *HNF4A* and *HNF4A-AS1*, *KIF12* and *COL27A1*, *F7* and *F10*, *SERPINF2* and *SERPINF1*, *RP11-116D2.1* and *APOB*, *ITIH1* and *ITIH3*, *C2orf72* and *RP11-223I10.1*, *HNF4A* and *RP5-881L22.6*, *CYP4F3* and *CYP4F12*, *THPO* and *CHRD*, *RP11-381N20.1* and *LINC01091*, *CCL15* and *CCL16*, *ISM1* and *RP5-1077I2.3*, *CCL16* and *RP11-104J23.1*, *RP11-713M15.1* and *SNTB1*, *COLCA1* and *POU2AF1*, *APOA1* and *APOA5*, *SYN3* and *TIMP3*, *CPVL* and *CHN2*, *PLCB1* and *PLCB1-IT1*, *UGT2B10* and *UGT2A3*, *CPVL* and *AC004593.3*, *COL27A1* and *ORM2*).

A

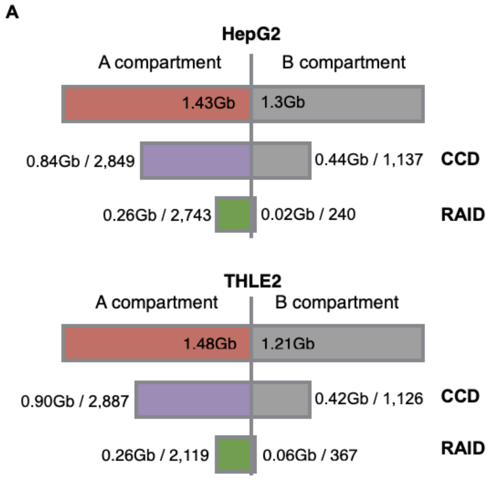

B

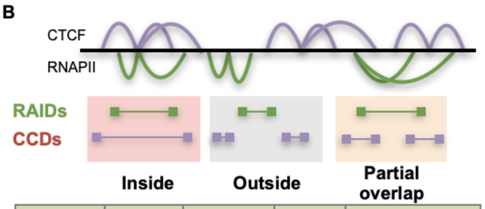

Cell line	Total RAIDs	In CCD	In gap	Overlapping CCD
HepG2	2,242	1,169	280	793
THLE2	3,099	1,567	359	1,182

C

	THLE2 vs HepG2	No. of HCC-specific genes (n=488)
change	B→A	71 (14.5%)
change	A→B	8
no change	A→A	252
no change	B→B	13
Other		42

	THLE2 vs HepG2	No. of HCC-suppressed genes (n=1,053)
change	B→A	48
change	A→B	150 (14.2%)
no change	A→A	562
no change	B→B	69
other		95

D

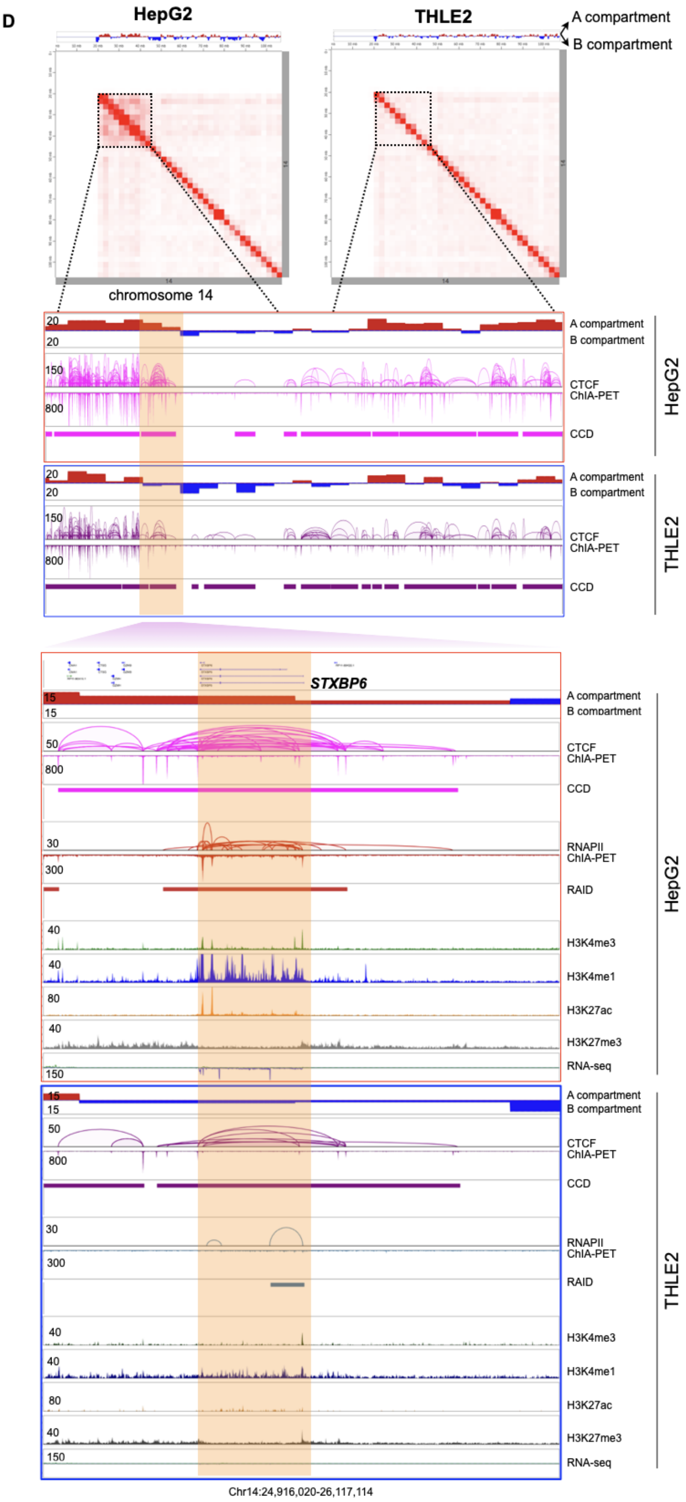

E

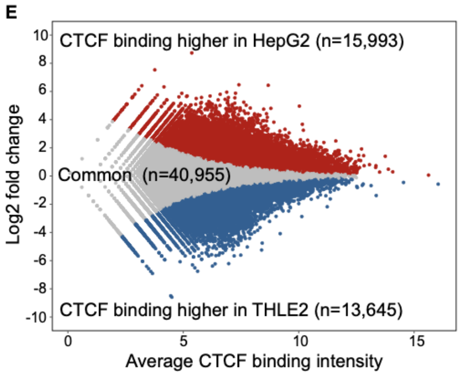

F

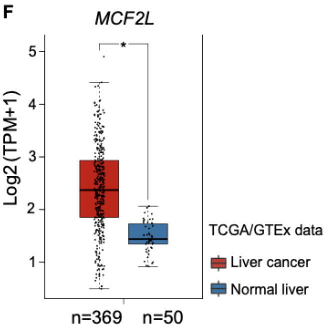

G

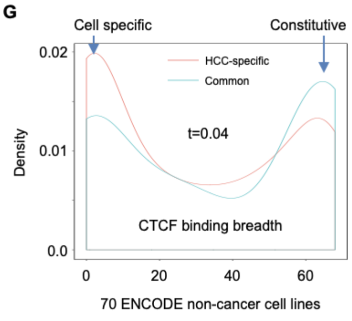

Figure S4. **Specific genome architecture links HCC-specific transcription.** Related to Figure 4.

A. Bar chart of genomic coverage by A/B compartments, CTCF-mediated chromatin contact domains (CCDs) and RAIDs in HepG2 and THLE2 cells. **B**. Schematic and statistics of Intersection relationship between CCDs and RAIDs in HepG2 and THLE2 cells. **C**. A/B compartment switch involving HCC-specific and HCC-suppressed genes in HepG2 (up panel) and THLE2 cells (low panel). **D**. An example of chromatin segment involved in A/B compartment switch with a HCC-specific gene (*STXBP6*) as viewed at different scales of genome organization by 2D contact profile and browser screenshots for A/B compartment, CTCF and RNAPII loops/peaks and domains (CCD and RAID). Tracks of histone ChIP-seq and RNA-seq are provided as epigenomic references. **E**. MA plot showing the comparison of the genome-wide CTCF binding peak intensity in HepG2 and THLE2 cells. Increased CTCF bindings in HepG2 (red), common CTCF binding (grey), and increased CTCF bindings in THLE2 (blue) are indicated. **F**. Gene expression of *MCF2L* was analyzed by GEPIA (http://gepia.cancer-pku.cn/) using TCGA-liver cancer (red) and TCGA normal+ GTEx liver (blue) RNA-seq data. TPM, transcripts per million. * $p < 0.01$, one-way ANOVA. **G**. CTCF binding breadth of HCC-specific gene associated CTCF binding sites (red curve) that are also showed increased binding in HepG2 over THLE2 and the common CTCF binding (green curve) that are shared in both HepG2 and THLE2 (p=0.04). CTCF binding sites in 70 ENCODE non-cancer cell lines were used for the breadth analysis (https://www.encodeproject.org). See also Supplemental Table 6.

A

	No. of CTCF binding sites	No. of CTCF peaks without SNP	No. of CTCF motifs without SNP	No. of CTCF peaks without indel	No. of CTCF motif without indel
TSS of HCC-specific genes	223 Higher in HepG2	218	223	190	221
TSS of HCC-suppressed genes	401 Higher in THLE2	401	401	401	401

B

	HepG2_rep1	HepG2_rep2	HepG2_rep3	THLE2_rep1
Source	This study	ENCODE	ENCODE	This study
Total Raw Reads	526 million	586 million	619 million	513 million
Total CpG sites (4x)	49.5 million	51.8 million	41.8 million	50 million

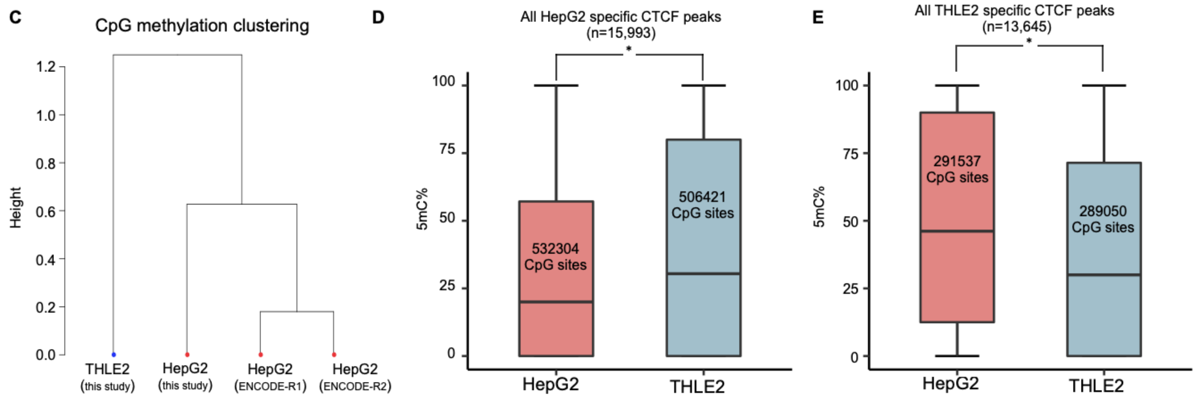

Figure S5. **Altered CTCF binding on the promoter is linked to CpG methylation on CTCF motif.** Related to Figure 5.

A. Statistics of genetic variations in CTCF peaks centered on the TSS (± 2 kb) of HepG2 specific genes with HepG2 increased CTCF binding and THLE2 specific genes with THLE2 increased CTCF binding. 25,079 liver/liver diseases related SNPs (the same as shown in Figure S2H) are used in this analysis. Mutations in the CTCF motif are identified via integration of whole genome sequencing data of HepG2 and THLE2. **B**. Summary of whole genome bisulfate sequencing QC metrix for in-house HepG2, THLE2 and ENCODE HepG2 data. **C**. Clustering analysis showing the correlation of in-house HepG2, THLE2 WGBS data with ENCODE HepG2 WGBS data. **D-E**. Box plots showing methylation level of all the HepG2 (D) and THLE2 (E) specific CTCF binding peaks in each cell line. (Wilcoxon Rank Sum Test. **p* value<0.01).

SUPPLEMENTAL TABLES

Supplementary Table 1: Dataset used in this study

**ChIA-PET**				
Cell line	Factor	Total reads	Both ends uniquely mapped reads	Source
HepG2	CTCF	191,792,776	16,926,897	This study (GSE144893)
THLE2		238,750,403	22375026	
HepG2	RNAPII	186,418,228	44,262,114	
THLE2		224,500,090	12368543	
**RNA-Seq**				
Cell line	Replicate	Total reads	Uniquely mapped reads	Source
HepG2	Rep1	65,073,204	38,743,516	ENCODE (GSM2072545)
	Rep2	69,179,706	43,600,754	ENCODE (GSM2072546)
THLE2	Rep1	138,436,208	100,830,182	This study (GSE144893)
	Rep2	147,856,630	107,865,330	This study (GSE144893)
**ChIP-Seq**				
Cell line	Factor	Total reads	Uniquely mapped reads	Source
HepG2	H3K4me1_Rep1	31,037,661	23,861,032	ENCODE (ENCLB695AMS)
	H3K4me1_Rep2	31,263,991	19,475,863	ENCODE (ENCLB695AMR)
THLE2	H3K4me1_Rep1	102,627,653	56,154,293	This study (GSE144893)
HpeG2	H3K4me3_Rep1	10,228,152	7,416,831	ENCODE(ENCLB695ABP)
	H3K4me3_Rep2	11,600,046	8,142,322	ENCODE(ENCLB695ABQ)
THLE2	H3K4me3_Rep1	51,474,079	33,844,197	This study (GSE144893)
HepG2	H3K27ac_Rep1	6,485,461	4,864,593	ENCODE(ENCLB695AQK)
	H3K27ac_Rep2	10,030,634	6,896,082	ENCODE (ENCLB695AQL)
THLE2	H3K27ac_Rep1	95,376,947	63,254,999	This study (GSE144893)
HepG2	H3K27me3_Rep1	35,229,601	20,931,524	ENCODE (ENCLB884PAC)
	H3K27me3_Rep2	11,726,880	5,432,917	ENCODE (ENCLB240EVZ)
THLE2	H3K27me3_Rep1	34,075,444	25,034,080	This study (GSE144893)
	H3K27me3_Rep2	32,604,871	20,553,395	This study (GSE144893)
HepG2	HNF4A	15,388,704	11,353,398	ENCODE (ENCSR000BLF)
	HNF4G	35,496,421	33,079,066	ENCODE (ENCSR000BNJ)
THLE2	HNF4A	35,088,507	22,246,019	This study (GSE144893)
	HNF4G	42,082,043	29,252,127	This study (GSE144893)
HepG2	Input	35,848,988	27,987,126	This study (GSE144893)
THLE2	Input	33,251,850	30,311,022	This study (GSE144893)
**Whole genome sequencing (WGS)**				
Cell line		Total reads	Uniquely mapped reads	Source
HepG2		636,581,233	588,102,880	This study (GSE144893)
THLE2		304,055,080	273,777,454	This study (GSE144893)
**Whole Genome Bisulfate Sequencing (WGBS)**				
Cell line	Replicate	Total reads	Uniquely mapped reads	Source
HepG2	Rep1	586,143,998	515,173,470	ENCODE (ENCLB223EJM)
HepG2	Rep2	618,935,892	221,416,274	ENCODE (ENCLB569VMN)
HepG2	Rep1	525,967,427	360,565,889	This study (GSE144893)
THLE2	Rep1	512,872,326	348,700,943	This study (GSE144893)

Supplementary Table 2: Liver cancer and normal liver marker genes

Gene list	Annotation		Reference in NCBI PubMed
AFP	Alpha fetoprotein	Cancer	PMID: 7684823
			PMID: 17187432
GPC3	Glypican 3	Cancer	PMID: 17006932
HULC	highly up-regulated in liver cancer	Cancer	PMID: 17241883
CCL15	Chemokine (C-C motif) ligand 15	Cancer	PMID: 26643668
CXCL2	Chemokine (C-X-C motif) ligand 2	Normal	PMID: 30293547
NNMT	Nicotinamide N-methyltransferase	Normal	PMID: 30093610
SAA1	Serum Amyloid A1	Normal	PMID: 17786358
SAA2	Serum Amyloid A2	Normal	PMID: 19171046

Supplementary Table 3: Expression breadth analysis data source

Cell/Tissue type	Source	ID	Assay
Cardiac_atrium_fibroblast	Encode	Encsr110bdy	Rna-seq
Cardiac_myocyte	Encode	Encsr379yae	Rna-seq
Cardiac_ventricle_fibroblast	Encode	Encsr369rvn	Rna-seq
Heart	Encode	Encsr000aez	Rna-seq
Pericardium_fibroblast	Encode	Encsr362hmx	Rna-seq
Regular_cardiac_myocyte	Encode	Encsr000aah	Rna-seq
Fibroblast_of_the_aortic_adventitia	Encode	Encsr000cuj	Rna-seq
Aortic_smooth_muscle_cell	Encode	Encsr000aaa	Rna-seq
Endothelial_cell_of_coronary_artery	Encode	Encsr000aaf	Rna-seq
Smooth_muscle_cell_of_the_coronary_artery	Encode	Encsr000aag	Rna-seq
Pericyte_cell	Encode	Encsr000ctx	Rna-seq
Thoracic_aorta_endothelial_cell	Encode	Encsr000cuk	Rna-seq
Vein_endothelial_cell	Encode	Encsr000cug	Rna-seq
Fibroblast_of_arm	Encode	Encsr797bpp	Rna-seq
Ht1080	Encode	Encsr535vtr	Rna-seq
Subcutaneous_preadipocyte	Encode	Encsr000cum	Rna-seq
Stomach	Encode	Encsr000afi	Rna-seq
Ht-29	Encode	Encsr971gpj	Rna-seq
Hematopoietic_multipotent_progenitor_cell	Encode	Encsr000cua	Rna-seq
Gm12878	Encode	Encsr000aed	Rna-seq
Karpas-422	Encode	Encsr314lxg	Rna-seq
Oci-ly7	Encode	Encsr001hhk	Rna-seq
K562	Encode	Encsr000aem	Rna-seq
Hepatocyte	Encode	Encsr908zas	Rna-seq
Hepg2	Encode	Encsr985kat	Rna-seq
Liver	Encode	Encsr000aeu	Rna-seq
Airway_epithelial_cell	Encode	Encsr822sug	Rna-seq
Epithelial_cell_of_alveolus_of_lung	Encode	Encsr897kto	Rna-seq
Bronchial_epithelial_cell	Encode	Encsr000aad	Rna-seq
Bronchial_smooth_muscle_cell	Encode	Encsr000aae	Rna-seq
Lung	Encode	Encsr000afc	Rna-seq
Bronchus_fibroblast_of_lung	Encode	Encsr620nsn	Rna-seq
Fibroblast_of_lung	Encode	Encsr000coo	Rna-seq
Lung_microvascular_endothelial_cell	Encode	Encsr000aap	Rna-seq
Nci-h460	Encode	Encsr164oct	Rna-seq
Smooth_muscle_cell_of_trachea	Encode	Encsr000aas	Rna-seq
Tracheal_epithelial_cell	Encode	Encsr000aar	Rna-seq
Pulmonary_artery_endothelial_cell	Encode	Encsr000aam	Rna-seq
Smooth_muscle_cell_of_the_pulmonary_artery	Encode	Encsr000aan	Rna-seq
Mammary_epithelial_cell	Encode	Encsr000cox	Rna-seq
Mammary_microvascular_endothelial_cell	Encode	Encsr815uvl	Rna-seq
Mcf-7	Encode	Encsr310fis	Rna-seq
Lhcn-m2	Encode	Encsr332dbs	Rna-seq
Myotube	Encode	Encsr828tei	Rna-seq
Sjcrh30	Encode	Encsr568yrp	Rna-seq
Skeletal_muscle_myoblast	Encode	Encsr000coy	Rna-seq
Skeletal_muscle_satellite_cell	Encode	Encsr000cui	Rna-seq
Skeletal_muscle_tissue	Encode	Encsr000aff	Rna-seq
Smooth_muscle_cell	Encode	Encsr052fja	Rna-seq
Articular_chondrocyte_of_knee_joint	Encode	Encsr000cue	Rna-seq
Mg63	Encode	Encsr971knw	Rna-seq
Osteoblast	Encode	Encsr000cuf	Rna-seq
Sjsa1	Encode	Encsr880ego	Rna-seq
A172	Encode	Encsr580gsx	Rna-seq
Astrocyte	Encode	Encsr233ijt	Rna-seq
H4	Encode	Encsr919qjt	Rna-seq
M059j	Encode	Encsr696smk	Rna-seq

Bipolar_spindle_neuron	Encode	Encsr225bbk	Rna-seq
Cerebellar_granule_cell	Encode	Encsr313iuo	Rna-seq
Cerebellum	Encode	Encsr000aew	Rna-seq
Daoy	Encode	Encsr254jjm	Rna-seq
Diencephalon	Encode	Encsr000aex	Rna-seq
Frontal_cortex	Encode	Encsr000aey	Rna-seq
Occipital_lobe	Encode	Encsr000afd	Rna-seq
Parietal_lobe	Encode	Encsr000afe	Rna-seq
Purkinje_cell	Encode	Encsr157nlf	Rna-seq
Pyramidal_cell	Encode	Encsr137rhz	Rna-seq
Spinal_cord	Encode	Encsr000afh	Rna-seq
Temporal_lobe	Encode	Encsr000afj	Rna-seq
Neural_cell	Encode	Encsr292tap	Rna-seq
Neural_progenitor_cell	Encode	Encsr244isq	Rna-seq
Camera-type_eye	Encode	Encsr000afo	Rna-seq
Tongue	Encode	Encsr000afl	Rna-seq
Nasal_cavity_respiratory_epithelium_epithelial_cell_of_viscerocranial_mucosa	Encode	Encsr000aal	Rna-seq
Sk-n-dz	Encode	Encsr136wgp	Rna-seq
Bladder_microvascular_endothelial_cell	Encode	Encsr000aab	Rna-seq
Smooth_muscle_cell_of_bladder	Encode	Encsr000aac	Rna-seq
Urinary_bladder	Encode	Encsr000aev	Rna-seq
Caki2	Encode	Encsr584jxd	Rna-seq
G401	Encode	Encsr653dfz	Rna-seq
Glomerular_endothelial_cell	Encode	Encsr878eut	Rna-seq
Kidney_epithelial_cell	Encode	Encsr373bdg	Rna-seq
Mesangial_cell	Encode	Encsr198tka	Rna-seq
Metanephros	Encode	Encsr000afa	Rna-seq
Epithelial_cell_of_proximal_tubule	Encode	Encsr118tvr	Rna-seq
Renal_cortical_epithelial_cell	Encode	Encsr000aaq	Rna-seq
Endometrial_microvascular_endothelial_cells	Encode	Encsr919mzm	Rna-seq
Myometrial_cell	Encode	Encsr371vgv	Rna-seq
Uterine_smooth_muscle_cell	Encode	Encsr000aav	Rna-seq
Uterus	Encode	Encsr000afn	Rna-seq
Pc-3	Encode	Encsr420nlc	Rna-seq
Skin_of_body	Encode	Encsr000afg	Rna-seq
Dermis_blood_vessel_endothelial_cell	Encode	Encsr000aai	Rna-seq
Fibroblast_of_dermis	Encode	Encsr000cuh	Rna-seq
Dermis_lymphatic_vessel_endothelial_cell	Encode	Encsr000aaj	Rna-seq
Dermis_microvascular_lymphatic_vessel_endothelial_cell	Encode	Encsr000aak	Rna-seq
Keratinocyte	Encode	Encsr000cpi	Rna-seq
Rpmi-7951	Encode	Encsr320brr	Rna-seq
Hair_follicle_dermal_papilla_cell	Encode	Encsr000cub	Rna-seq
Hair_follicular_keratinocyte	Encode	Encsr680use	Rna-seq
A375	Encode	Encsr504vxc	Rna-seq
Sk-mel-5	Encode	Encsr669kqu	Rna-seq
Melanocyte_of_skin	Encode	Encsr000cuq	Rna-seq
H7-hesc	Encode	Encsr490sqh	Rna-seq
Induced_pluripotent_stem_cell	Encode	Encsr368qpc	Rna-seq
Thyroid_gland	Encode	Encsr000afk	Rna-seq
Placental_epithelial_cell	Encode	Encsr000cup	Rna-seq
Epithelial_cell_of_umbilical_artery	Encode	Encsr000aat	Rna-seq
Smooth_muscle_cell_of_the_umbilical_artery	Encode	Encsr000aau	Rna-seq
Umbilical_cord	Encode	Encsr000afm	Rna-seq
Endothelial_cell_of_umbilical_vein	Encode	Encsr000coz	Rna-seq
Fibroblast_of_villous_mesenchyme	Encode	Encsr000cul	Rna-seq

Supplementary Table 4: Statistics of RNAPII-associated chromatin interactions

**Interactions**	**P-P**	**E-P**	**E-E**	
**Common**	1,415	369	54	Statistics of RNAPII associated chromatin interaction
**HepG2 specific**	6,021	6,241	2,738	
**THLE2 specific**	3,674	5342	2,445	
	HepG2	THLE2	Statistics of expressed genes in SG and MG domains	
**MGs (genes)**	1,265 (6,436)	1,571 (6,386)		
**SGs (genes)**	509 (509)	916 (916)		
			Statistics of upregulated and downregulated genes in SG and MG domains	
	HepG2	THLE2		
**MGs (genes)**	165 (196)	306 (347)		
**SGs (genes)**	40 (40)	144 (144)		

Supplementary Table 5: HepG2 ENCODE TF ChIP-Seq data source

Biosample	Source	Accession	Assay type	Target of TF
HepG2	ENCODE	ENCFF377SDH	ChIP-seq	AGO1
		ENCFF848FVO	ChIP-seq	AGO2
		ENCFF785LTB	ChIP-seq	ARID3A
		ENCFF357FTD	ChIP-seq	ARNT
		ENCFF835XXH	ChIP-seq	ASH2L
		ENCFF215TZZ	ChIP-seq	ATF2
		ENCFF015XMO	ChIP-seq	ATF3
		ENCFF510UKU	ChIP-seq	ATF7
		ENCFF465OFU	ChIP-seq	ATM
		ENCFF413KUZ	ChIP-seq	BCLAF1
		ENCFF502XEV	ChIP-seq	BHLHE40
		ENCFF619ZLP	ChIP-seq	BRCA1
		ENCFF367CAC	ChIP-seq	BRD4
		ENCFF209ALR	ChIP-seq	CBX2
		ENCFF478NLQ	ChIP-seq	CCAR2
		ENCFF419CGD	ChIP-seq	CEBPB
		ENCFF684MOG	ChIP-seq	CEBPZ
		ENCFF553QDJ	ChIP-seq	CHD2
		ENCFF346PKZ	ChIP-seq	CHD4
		ENCFF835UPN	ChIP-seq	CREB1
		ENCFF569NVH	ChIP-seq	CREM
		ENCFF329MOF	ChIP-seq	CTCF
		ENCFF234MZM	ChIP-seq	EHMT2
		ENCFF786XDZ	ChIP-seq	ELF1
		ENCFF811CMJ	ChIP-seq	EP300
		ENCFF653JVV	ChIP-seq	ETS1
		ENCFF816PYF	ChIP-seq	ETV4
		ENCFF011DOP	ChIP-seq	FIP1L1
		ENCFF105NJB	ChIP-seq	FOSL2
		ENCFF722GFS	ChIP-seq	FOXA1
		ENCFF459HJZ	ChIP-seq	FOXA2
		ENCFF851TZY	ChIP-seq	FOXK2
		ENCFF304CJQ	ChIP-seq	FOXP1
		ENCFF383WAL	ChIP-seq	GABPA
		ENCFF081YHC	ChIP-seq	GATA4
		ENCFF224MFN	ChIP-seq	GTF2F1
		ENCFF125RMF	ChIP-seq	HCFC1
		ENCFF743SDK	ChIP-seq	HDAC1
		ENCFF138UQY	ChIP-seq	HDAC2
		ENCFF964PGC	ChIP-seq	HDAC6
		ENCFF044GIB	ChIP-seq	HNF1A
		ENCFF222YCT	ChIP-seq	HNF4A
		ENCFF267YOX	ChIP-seq	HNF4G
		ENCFF506GGI	ChIP-seq	HNRNPH1
		ENCFF899PGY	ChIP-seq	HNRNPK
		ENCFF866NDX	ChIP-seq	HNRNPL
		ENCFF026ZCX	ChIP-seq	HNRNPLL
		ENCFF373QRK	ChIP-seq	HNRNPUL1
		ENCFF157FKT	ChIP-seq	IKZF1
		ENCFF596XCU	ChIP-seq	JUND
		ENCFF913DIG	ChIP-seq	KAT2B
		ENCFF568WND	ChIP-seq	KDM1A
		ENCFF243FMU	ChIP-seq	KDM5A
		ENCFF914THM	ChIP-seq	LCORL
		ENCFF645NAR	ChIP-seq	MAFF
		ENCFF814UDQ	ChIP-seq	MAFK
		ENCFF999ZXB	ChIP-seq	MAX
		ENCFF762QHA	ChIP-seq	MAZ
		ENCFF634BXB	ChIP-seq	MNT
		ENCFF138JAZ	ChIP-seq	NBN
		ENCFF193YSE	ChIP-seq	NCOR1
		ENCFF266HXY	ChIP-seq	NFE2L2
		ENCFF862BRX	ChIP-seq	NFRKB

Biosample	Source	Accession	Assay type	Target
HepG2	ENCODE	ENCFF517JWG	ChIP-seq	NONO
		ENCFF487OSZ	ChIP-seq	NR2F6
		ENCFF920ITW	ChIP-seq	NRF1
		ENCFF180HWU	ChIP-seq	PCBP1
		ENCFF085ENX	ChIP-seq	PHB2
		ENCFF327PCB	ChIP-seq	PHF8
		ENCFF014AER	ChIP-seq	PLRG1
		ENCFF116TSU	ChIP-seq	POLR2A
		ENCFF783DEP	ChIP-seq	POLR2AphosphoS2
		ENCFF979IOQ	ChIP-seq	POLR2AphosphoS5
		ENCFF733YFT	ChIP-seq	POLR2G
		ENCFF385HAD	ChIP-seq	PRPF4
		ENCFF623DYE	ChIP-seq	PTBP1
		ENCFF061QPF	ChIP-seq	RAD21
		ENCFF353OUV	ChIP-seq	RAD51
		ENCFF390PNX	ChIP-seq	RBFOX2
		ENCFF298ESK	ChIP-seq	RCOR1
		ENCFF618IBW	ChIP-seq	REST
		ENCFF076BOS	ChIP-seq	RFX1
		ENCFF645MNT	ChIP-seq	RFX5
		ENCFF846ZLV	ChIP-seq	RNF2
		ENCFF799DNN	ChIP-seq	RXRA
		ENCFF588MIJ	ChIP-seq	SIN3A
		ENCFF229MLS	ChIP-seq	SIN3B
		ENCFF583YAO	ChIP-seq	SKI
		ENCFF840XVN	ChIP-seq	SMARCC2
		ENCFF755URS	ChIP-seq	SMARCE1
		ENCFF346SQV	ChIP-seq	SMC3
		ENCFF371FIK	ChIP-seq	SNRNP70
		ENCFF860FKS	ChIP-seq	SOX13
		ENCFF425UQS	ChIP-seq	SOX6
		ENCFF330CLZ	ChIP-seq	SP1
		ENCFF083OZJ	ChIP-seq	SRSF4
		ENCFF284HXN	ChIP-seq	SRSF9
		ENCFF602ZTI	ChIP-seq	SUZ12
		ENCFF575XQE	ChIP-seq	TAF1
		ENCFF843YBE	ChIP-seq	TARDBP
		ENCFF144VBS	ChIP-seq	TBL1XR1
		ENCFF425TFY	ChIP-seq	TBP
		ENCFF852LYH	ChIP-seq	TBX3
		ENCFF997NWM	ChIP-seq	TCF12
		ENCFF164KYP	ChIP-seq	TCF7
		ENCFF263VIF	ChIP-seq	TFAP4
		ENCFF985NVN	ChIP-seq	TRIM22
		ENCFF749HXW	ChIP-seq	U2AF1
		ENCFF801SLM	ChIP-seq	U2AF2
		ENCFF962DEO	ChIP-seq	USF1
		ENCFF837IBY	ChIP-seq	XRCC5
		ENCFF566DXN	ChIP-seq	YBX1
		ENCFF858QVH	ChIP-seq	YY1
		ENCFF697VUK	ChIP-seq	ZBTB33
		ENCFF791BSP	ChIP-seq	ZBTB40
		ENCFF363GGS	ChIP-seq	ZBTB7A
		ENCFF881THB	ChIP-seq	ZFP36
		ENCFF918ZBN	ChIP-seq	ZHX2
		ENCFF448GYO	ChIP-seq	ZKSCAN1
		ENCFF438HPL	ChIP-seq	ZMYM3
		ENCFF139NXQ	ChIP-seq	ZNF207
		ENCFF217YYT	ChIP-seq	ZNF24
		ENCFF672RYT	ChIP-seq	ZNF282
		ENCFF145TSQ	ChIP-seq	ZNF384

Supplementary Table 6: Source of CTCF data used for breadth analysis

Biosample	Source	Accession	Assay type	Target
GM12878	ENCODE	ENCFF002DAJ	ChIP-seq	CTCF
endothelial_cell_of_umbilical_vein	ENCODE	ENCFF002DBA	ChIP-seq	CTCF
K562	ENCODE	ENCFF002DBD	ChIP-seq	CTCF
GM12801	ENCODE	ENCFF002DCF	ChIP-seq	CTCF
foreskin_fibroblast	ENCODE	ENCFF002DCY	ChIP-seq	CTCF
HL-60	ENCODE	ENCFF002DDA	ChIP-seq	CTCF
fibroblast_of_lung	ENCODE	ENCFF002DDO	ChIP-seq	CTCF
22Rv1	ENCODE	ENCFF030BPR	ChIP-seq	CTCF
PC-9	ENCODE	ENCFF032DEW	ChIP-seq	CTCF
brain_microvascular_endothelial_cell	ENCODE	ENCFF065LHJ	ChIP-seq	CTCF
neural_progenitor_cell	ENCODE	ENCFF072ZEU	ChIP-seq	CTCF
SU-DHL-6	ENCODE	ENCFF099NBE	ChIP-seq	CTCF
GM12865	ENCODE	ENCFF132AJR	ChIP-seq	CTCF
retinal_pigment_epithelial_cell	ENCODE	ENCFF139DOR	ChIP-seq	CTCF
LNCAP	ENCODE	ENCFF155SPQ	ChIP-seq	CTCF
kidney_epithelial_cell	ENCODE	ENCFF168VBK	ChIP-seq	CTCF
MCF-7	ENCODE	ENCFF176YNV	ChIP-seq	CTCF
GM10248	ENCODE	ENCFF210PLK	ChIP-seq	CTCF
keratinocyte	ENCODE	ENCFF226MQR	ChIP-seq	CTCF
RWPE1	ENCODE	ENCFF241JKV	ChIP-seq	CTCF
GM20000	ENCODE	ENCFF244DQW	ChIP-seq	CTCF
AG09319	ENCODE	ENCFF245PXW	ChIP-seq	CTCF
OCI-LY1	ENCODE	ENCFF247GZH	ChIP-seq	CTCF
PC-3	ENCODE	ENCFF258RWN	ChIP-seq	CTCF
cardiac_muscle_cell	ENCODE	ENCFF266GGD	ChIP-seq	CTCF
HFF-Myc	ENCODE	ENCFF282QFS	ChIP-seq	CTCF
mammary_epithelial_cell	ENCODE	ENCFF288RFS	ChIP-seq	CTCF
GM12873	ENCODE	ENCFF295AWO	ChIP-seq	CTCF
Ishikawa	ENCODE	ENCFF296XCF	ChIP-seq	CTCF
VCaP	ENCODE	ENCFF304IDM	ChIP-seq	CTCF
astrocyte_of_the_spinal_cord	ENCODE	ENCFF312HCK	ChIP-seq	CTCF
HEK293	ENCODE	ENCFF328FUO	ChIP-seq	CTCF
A549	ENCODE	ENCFF335GSE	ChIP-seq	CTCF
foreskin_fibroblast	ENCODE	ENCFF337WIE	ChIP-seq	CTCF
bipolar_neuron	ENCODE	ENCFF355OQM	ChIP-seq	CTCF
neutrophil	ENCODE	ENCFF374BNP	ChIP-seq	CTCF
bronchial_epithelial_cell	ENCODE	ENCFF401ONY	ChIP-seq	CTCF
H1-hESC	ENCODE	ENCFF402JJK	ChIP-seq	CTCF
SK-N-SH	ENCODE	ENCFF403SBB	ChIP-seq	CTCF
astrocyte	ENCODE	ENCFF415WKV	ChIP-seq	CTCF
HCT116	ENCODE	ENCFF418WAW	ChIP-seq	CTCF
DOHH2	ENCODE	ENCFF421PBO	ChIP-seq	CTCF
CD14-positive_monocyte	ENCODE	ENCFF437LHG	ChIP-seq	CTCF
fibroblast_of_mammary_gland	ENCODE	ENCFF438XHB	ChIP-seq	CTCF
GM13977	ENCODE	ENCFF445ATY	ChIP-seq	CTCF
GM12874	ENCODE	ENCFF446MKT	ChIP-seq	CTCF
myotube	ENCODE	ENCFF448MBM	ChIP-seq	CTCF
B_cell	ENCODE	ENCFF449NOT	ChIP-seq	CTCF
Panc1	ENCODE	ENCFF450CNK	ChIP-seq	CTCF
IMR-90	ENCODE	ENCFF453XKM	ChIP-seq	CTCF
foreskin_keratinocyte	ENCODE	ENCFF459YEI	ChIP-seq	CTCF
epithelial_cell_of_proximal_tubule	ENCODE	ENCFF470PNM	ChIP-seq	CTCF
epithelial_cell_of_esophagus	ENCODE	ENCFF528VFN	ChIP-seq	CTCF
fibroblast_of_lung	ENCODE	ENCFF570FLB	ChIP-seq	CTCF
smooth_muscle_cell	ENCODE	ENCFF586TYX	ChIP-seq	CTCF
osteoblast	ENCODE	ENCFF609ETO	ChIP-seq	CTCF
neural_cell	ENCODE	ENCFF618DDO	ChIP-seq	CTCF
AG09309	ENCODE	ENCFF622DUX	ChIP-seq	CTCF
GM13976	ENCODE	ENCFF622GQO	ChIP-seq	CTCF
fibroblast_of_dermis	ENCODE	ENCFF638OUB	ChIP-seq	CTCF
BJ	ENCODE	ENCFF649IRT	ChIP-seq	CTCF
OCI-LY3	ENCODE	ENCFF653ZMJ	ChIP-seq	CTCF
C4-2B	ENCODE	ENCFF658MKE	ChIP-seq	CTCF
astrocyte_of_the_cerebellum	ENCODE	ENCFF660HHS	ChIP-seq	CTCF

KMS-11	ENCODE	ENCFF662IOC	ChIP-seq	CTCF
GM12864	ENCODE	ENCFF671RVI	ChIP-seq	CTCF
AG04450	ENCODE	ENCFF681OWQ	ChIP-seq	CTCF
liver	ENCODE	ENCFF690BYG	ChIP-seq	CTCF
lung	ENCODE	ENCFF694SEF	ChIP-seq	CTCF
epithelial_cell_of_prostate	ENCODE	ENCFF698WAX	ChIP-seq	CTCF
choroid_plexus_epithelial_cell	ENCODE	ENCFF700ILD	ChIP-seq	CTCF
GM12875	ENCODE	ENCFF733YSO	ChIP-seq	CTCF
fibroblast_of_lung	ENCODE	ENCFF734DZF	ChIP-seq	CTCF
fibroblast_of_villous_mesenchyme	ENCODE	ENCFF738CXX	ChIP-seq	CTCF
AG10803	ENCODE	ENCFF746CIL	ChIP-seq	CTCF
GM23338	ENCODE	ENCFF765BRD	ChIP-seq	CTCF
HeLa-S3	ENCODE	ENCFF768OPK	ChIP-seq	CTCF
hepatocyte	ENCODE	ENCFF776XRJ	ChIP-seq	CTCF
MM.1S	ENCODE	ENCFF782KFG	ChIP-seq	CTCF
RWPE2	ENCODE	ENCFF788CHY	ChIP-seq	CTCF
GM10266	ENCODE	ENCFF855HSK	ChIP-seq	CTCF
fibroblast_of_the_aortic_adventitia	ENCODE	ENCFF859PRV	ChIP-seq	CTCF
SK-N-SH	ENCODE	ENCFF861DPF	ChIP-seq	CTCF
OCI-LY7	ENCODE	ENCFF862VFJ	ChIP-seq	CTCF
pancreas	ENCODE	ENCFF867ERM	ChIP-seq	CTCF
medulloblastoma	ENCODE	ENCFF874DDR	ChIP-seq	CTCF
spleen	ENCODE	ENCFF881YDR	ChIP-seq	CTCF
Caco-2	ENCODE	ENCFF882YQH	ChIP-seq	CTCF
T47D	ENCODE	ENCFF903ZMF	ChIP-seq	CTCF
H54	ENCODE	ENCFF909JAZ	ChIP-seq	CTCF
HCT116	ENCODE	ENCFF917ZPO	ChIP-seq	CTCF
WERI-Rb-1	ENCODE	ENCFF924OMX	ChIP-seq	CTCF
NCI-H929	ENCODE	ENCFF924XFS	ChIP-seq	CTCF
GM06990	ENCODE	ENCFF930QUM	ChIP-seq	CTCF
fibroblast_of_pulmonary_artery	ENCODE	ENCFF933VBD	ChIP-seq	CTCF
A673	ENCODE	ENCFF947IJX	ChIP-seq	CTCF
BE2C	ENCODE	ENCFF965BBC	ChIP-seq	CTCF
AG04449	ENCODE	ENCFF970AVM	ChIP-seq	CTCF
LNCaP_clone_FGC	ENCODE	ENCFF988IOQ	ChIP-seq	CTCF

SUPPLEMENTAL METHODS

I. EXPERIMENTAL METHODS

Cell Culture

HepG2 (ATCC® HB-8065™) and THLE2 (ATCC® CRL-2706™) cell lines were purchased from ATCC. HepG2 cells were cultured in DMEM supplemented with 10% FBS and 100 U/mL penicillin/streptomycin. THLE2 cells were cultured by using BEGM Bullet Kit (Lonza/Clonetics Corporation, cat.#CC3170). The culture dish used for THLE2 were precoated with a mixture of 0.01 mg/mL fibronectin, 0.03 mg/mL bovine collagen type I and 0.01 mg/mL bovine serum albumin dissolved in BEBM medium.

Long-reads ChIA-PET

Long Reads ChIA-PET libraries with antibody against RNAPII and CTCF were generated with HepG2 and THLE2 cells by following the ChIA-PET protocol reported previously(14,43). Briefly, when the cells were grown to 80% confluency, 40 ml of ethylene glycol bis (EGS)(Thermo Fisher Scientific, cat.#21565)/1 X PBS solution was added to each culture dish followed by shaking on orbital shaker for 45 min at room temperature (RT). After that, 1.1 mL of 37% formaldehyde (final concentration: 1%) (EMD Millipore, cat. # 344198) was added to each dish followed by shaking on orbital shaker for 20 min at RT. Subsequently, 3.57 mL of 2.5 M glycine (final concentrate: 0.2 M)(Sigma-Aldrich, cat# G8898) was added to each dish followed by shaking on orbital shaker for 10 min at RT. The cells were scraped off the dish by cell scraper and transferred into 50 ml corning tube and then centrifuged at 2000 rpm for 10min, 4°C. Media was discarded by pipetting and the cells were washed for twice by adding 20 mL chilled PBS and then centrifuged at 2000 rpm for 5 min, 4°C. After removing the supernatant, the cells pellets were stored at -80°C.

300 millions of fixed HepG2/THLE2 cells were used to generate one ChIA-PET library. Briefly, after cell lysis and nuclear lysis, the nucleus was sonicated into ~1 kb size. Then chromatin was precleared using protein G beads and immunoprecipitation was performed using anti-RNAPII (BioLegend, cat.# 8WG16)/anti-CTCF antibody (Abcam, cat.# ab70303) coated protein G beads (Life Technologies, cat. no. 10009D). Then on-beads A-tailing was performed using Klenow Fragment (3′>5′ exo-) (NEB, cat.#M0212L) and dATP (100 mM; NEB, cat.#N0440S). On-bead proximity ligation was performed using in-house bridge linker (F: 5′- /5Phos/CGCGATATC/iBIOdT/TATCTGACT -3′, R: 5′- /5Phos/GTCAGATAAGATATCGCGT -3′. HPLC purified, from Integrated DNA Technologies) and T4 DNA ligase (Thermo Fisher Scientific, cat.#EL0013). After that, proximity ligated chromatin complexes were eluted from beads and proteinase K (Life Technologies, cat.#AM2548) was added to reverse cross-linked chromatin complexes for overnight. After Phenol:chloroform:IAA (Ambion, cat.#AM9730) extraction and isopropanol precipitation, tagmentation was performed on the proximity-ligated DNA by Tn5 transposase from Nextera DNA Sample Prep Kit (Illumina, cat.#FC-121-1031). Then tagmented DNA was immobilized on M280 streptavidin dynabeads (Invitrogen, cat.#11205D). PCR amplification was performed on beads using Nextera DNA Sample Prep Kit and the products were purified by AMPure XP beads (Beckman, cat.#A63881) and subjected to size selection (300-600 bp) on BluePippin instrument (Sage Science) using Blue Pippin Cassette Kit (Sage Science, cat.#BDF2010). DNA library was sequenced on Illumina Nextseq 500 by paired-end 150 bp.

ChIP-seq

10 millions of HepG2 and THLE2 cells were fixed by adding 1% (final concentration) formaldehyde (EMD Millipore, cat# 344198). Fixation was stopped by the addition of 0.125 M glycine (final concentration)(Sigma-Aldrich, cat# G8898). Chromatin was isolated by the addition of lysis buffer and lysates were sonicated and the DNA sheared to an average length of 200-300 bp by Bioruptor(Diagenode). Genomic DNA (Input) was prepared by treating aliquots of chromatin with proteinase K (Life Technologies, cat.#AM2548) at 65°C

for de-crosslinking overnight, followed by purification using MinElute PCR Purification Kit (Qiagen, cat.# 28004). Sonicated chromatin was precipitated by H3K4me1 (Abcam, cat.#ab8895), H3K4me3 (Abcam, cat.#ab8580), H3K27Ac (Abcam, cat.#ab4729), H3K27me3 (Millipore, cat.#07-449) antibodies. Precipitated chromatin complexes were washed, eluted from the beads with SDS buffer, and subjected to proteinase K treatment. De-crosslinking was performed by incubation overnight at 65°C. ChIPed DNA was purified by Qiagen MinElute kit. Library preparation was performed using the KAPA Hyper Prep kit (Roche, cat.#07962363001) and sequenced on Illumina Nextseq 500 by single-end 50 bp.

RNA-seq

Total RNA was extracted from HepG2 and THLE2 using PureLink® RNA Mini kit (Thermo Scientific, cat.#12183020) and subjected to on-column DNase I (Thermo Scientific, cat.#18068015) treatment. 1 µg RNA was performed ribosomal RNA depletion using the riboZero rRNA removal kit (Illumina, cat.# MRZH11124). RNA was fragmented into 200 bp in 5X NEBNext First Strand Synthesis Reaction Buffer (New England BioLabs, cat.#E7525S). First strand cDNA was synthesized using random hexamer primer and M-MuLV Reverse Transcriptase (New England BioLabs, cat.#M0253S). Second strand cDNA synthesis was subsequently performed using DNA Polymerase I (New England BioLabs, cat.# M0209S) and RNase H (New England BioLabs, cat.# M0297S). Library preparation was performed using the KAPA Hyper Prep kit (Roche, cat.#07962363001). DNA library was sequenced on Hiseq 2500 by paired- end 100 bp.

Whole Genome Sequencing

A total amount of 2 µg DNA per sample (HepG2/THLE2) was used as input material for the DNA library preparations. Sequencing library was generated using Truseq Nano DNA HT Sample Prep Kit (Illumina) as per manufacturer's instruction and index codes were added to each sample. Briefly, genomic DNA sample was fragmented by sonication to a size of 300-500 bp by Covaris M220. Then DNA fragments were endpolished, A-tailed, and ligated with

the full-length adapter for Illumina sequencing, followed by further PCR amplification. After PCR products were purified using AMPure beads, libraries were analyzed for size distribution by Agilent 2100 Bioanalyzer and quantified using KAPA Hyper Prep kit (Roche, cat.#07962363001) and sequenced on Illumina HiSeqXten by pair-end 150 bp.

Whole Genome Bisulfate Sequencing

2 μg of HepG2/THLE2 genomic DNA was sonicated using a Covaris M220 into a size of 300-500 bp. Sodium bisulfite conversion of all DNA samples was performed using the EZ DNA Methylation kit (Zymo EZ DNA Methylation Kit, Zymo Research). All libraries were subjected to quality control by Agilent Bioanalyzer examination and quantified using the KAPA Hyper Prep kit (Roche, cat.#07962363001) and sequenced on Illumina HiSeqXten by pair-end 150 bp.

II. QUANTIFICATION AND STATISTICAL ANALYSIS

ChIA-PET data processing

We used an in-house pipeline (Ref) to process ChIA-PET data, details are as follows:

1. Linker filtering and read alignment:

The input file of the ChIA-PET sequencing data is **fastq.gz**. Briefly, paired-end tag (PET) read sequences were scanned for the bridge linker sequence and only PETs with the bridge linker were retained for downstream processing. After trimming the linkers, the flanking sequences were mapped to the human reference genome (hg19) using a hybrid of BWA-MEM and BWA-ALN, and only uniquely aligned (MAPQ ≥ 30) PETs were retained. PCR duplicates were removed. The BAM file of PET reads is now ready for further analysis.

2. PET clustering:

The BAM file of PET reads are from three categories: (a) PET reads with no linker sequence detected, (b) PET reads with a linker sequence detected but with only one usable genomic tag and (c) PET reads with a linker sequence detected with both ends having genomic tags. Only PET reads in (c) are used for further detecting interaction loops.

Each PET in (c) was categorized as either a self-ligation PET (two ends of the same DNA fragment) or inter-ligation PET (two ends from two different DNA fragments in the same chromatin complex) by evaluating the genomic span between the two ends of a PET. PETs with a genomic span less than or equal to 8 kb are classified as self-ligation PETs and are

used as a proxy for ChIP fragments since they are derived in a manner analogous to ChIP-seq mapping for protein binding sites. PETs with a genomic span greater than 8 kb are classified as inter-ligation PETs and represent the long-range interactions of interest. To be more representative of the interacting chromatin fragments, the 5' end of each inter-ligation PET was extended by 500 bp along the reference genome. To reflect the frequency of interaction between two loci, the extended inter-ligation PETs that directly overlapped were classified as one PET cluster. The PET counts in a PET cluster reflects the relative frequency of interaction between two genomic regions. It was observed that many anchors of distinct PET clusters were located within the same protein binding peak. These binding peaks reflect the real chromatin interaction loci in the nucleus. In order to streamline the PET clusters data structure, we collapsed the individual anchors of all PET clusters with 500 bp extensions to generate merged anchors. For anchors with overlapped binding peaks, we used the summit as the centers of interacting loci. We referred the merged PET clusters as chromatin interaction loops. Un-clustered individual inter-ligation PETs and PETs in the clusters below the PET cutoff are referred as PET singletons.

3. Peak calling:

All uniquely mapped and non-redundant analysis-ready reads including self-ligation and inter-ligation are used for identifying protein binding peaks. In addition, two categories of PET reads that were excluded from chromatin loop detection are recovered and included in peak-calling for protein binding. Specifically, the recovered read categories are (a) PET reads with no linker sequence detected and (b) PET reads with a linker sequence detected but with only one usable genomic tag. While these reads are uninformative for chromatin loop detection, they can be informative for peak-calling of protein binding.

Peak calling for protein binding is then performed using MACS2 (https://hbctraining.github.io/Intro-to-ChIPseq/lessons/05_peak_calling_macs.html). In addition, bedtools[1] is used to generate BedGraph files of the protein factor binding coverage along the chromosomes for browser-based visualization.

RNA-Seq analysis

1. Quality control and mapping

Quality control analysis was performed using FastQC (https://www.bioinformatics.babraham.ac.uk/projects/fastqc/) and Trimmomatic[2] with the parameters: HEADCROP:10 SLIDINGWINDOW:4:15 MINLEN:80. Reads were mapped to hg19 reference genome using STAR[3] with default parameters. Reads with mapping quality score less than 30 were filtered. FeatureCounts[4] were used to count the number of reads mapped to each gene based on GENCODE version 14.

2. Differentially expressed genes (DEGs)

Differentially expressed genes (DEGs) were identified using DESeq2[5] with the cutoff: $\log_2$ fold change ≥ 4 or ≤-4, p value≤ 0.0001. DEGs are then referred to as upregulated genes (HCC-specific) and downregulated genes in HepG2. We used MA plot to visualize upregulated and downregulated genes in two cell lines and labelled some maker genes.

3. Maker genes expression in LIHC samples

Four upregulated genes (*GPC3*, *AFP*, *ACSM1* and *APOC2*) and four downregulated genes (*SAA1*, *SAA2*, *NNMT* and *CXCL2*) were selected for expression comparison between liver cancer and normal liver samples from TCGA and GTEx respectively using the web server GEPIA (http://gepia.cancer-pku.cn/).

Expression breadth analysis

Total RNA-seq data for 112 human tissues/cells with genes annotated by GENOCDE version 19 were downloaded from https://www.encodeproject.org/data/annotations/v2/. We then calculated the number of tissue/cell types (expression breadth) for upregulated, downregulated and other genes expressed in with expression cutoff TPM >=1. Other genes were used as control. The p-value is calculated using the nonparametric Kolmogorov-Smirnov test.

4. Filtering the genes with low expression and low RNAPII binding

In this study, we only focused on protein-coding and lincRNAs genes as they have independent TSSs. We further identified a set of "expressed genes" for both HepG2 and THLE2 after plotting expression level distribution for all the protein-coding and lincRNA genes and filtering some extremely low expressed genes. We used the cutoff ($\log_2$ fold change >4 or <4 and qvalue > 0.0001) to identify differentially expressed genes (referred to upregulated and downregulated genes). Upregulated and downregulated genes without RNAPII peaks around their promoter regions (+/- 2 kb around TSSs) will be filtered for downstream analysis.

ChIP-seq analysis

Reads were mapped to reference genome using BWA-MEM[6]. PCR duplicates were removed using Picard software (https://broadinstitute.github.io/picard/). Reads with mapping quality score less than 20 were filtered. Binding peaks were called using MACS2[7] with the following parameters: --broad –nomodel.

Chromatin interaction loops analysis

ChIA-PET loops reflect interactions between two or multiple protein binding sites. Thus, we only calculated the interaction anchors with peak support. For RNAPII ChIA-PET data, at least one anchor has peak support as some enhancers have low RNAPII binding

signal which may not be detected by MACS2. For CTCF ChIA-PET data, both anchors should have peak support.

Identification of CTCF-mediated chromatin contact domains (CCDs) and RNAPII associated interacting domain (RAID)

We identified CTCF-mediated chromatin contact domains (CCDs) as previously described (Tang et al., 2015) and RNAPII associated interacting domains (RAIDs) based on similar criteria using CTCF and RNAPII ChIA-PET data in both HepG2 and THLE2 cell type, respectively. Firstly, in each chromosome interconnected chromatin loops (CTCF or RNAPII-associated loops) that are formed in daisy chains and covered candidate domain regions based on the continuous connectivity are considered as a chromatin interaction domain, usually covering multiple chromatin elements such as insulator, enhancer, and promoters. The rest of the genome with no CTCF or RNAPII loop coverage is considered as gap regions. Secondly, to further refine the contact domains, we calculated the aggregated CTCF or RNAPII loop coverage along all chromosomes at base-pair resolution and identified the regions with very low loop coverage (lower than the 5th percentile), which were subtracted for downstream analysis. Finally, the resulted chromatins domains with genomic size smaller than 10 kb are excluded from the downstream analyses.

For HepG2 and THLE2, we identified 2,385 and 3,317 CCDs and 2,242 and 3,099 RAIDs in HepG2 and THLE2 respectively.

Identification of Transcription Models from RNAPII associated Chromatin Interactions

Based on chromatin interactions and RNAPII binding signals, genes can be classified into three different transcription models with distinct genomic properties: basal promoters model (without interaction but bound by RNAPII), single-gene model (TSS interact with enhancers but not promoters), multi-gene model (TSS not only interact with enhancers but also interact with the promoter of other genes). We assigned ChIA-PET RNAPII anchors to promoters or enhancers and then treated the whole interactome as a network. After we identified multi-gene complex, we calculated the portion of up- and down-regulated genes in MG complex and whether two or more up- or down-regulated genes showed up together in a single MG.

Identification of enhancers and enhancer-like promoters

Enhancers were identified using three available histone marks: H3K4me1 (general enhancer mark), H3K27ac (active regulatory elements mark) and H3K27me3 (repressed regulatory elements mark). We divide 2.5 kb window around H3K4me1 peak summit into 20 equally size bins and then calculate number of H3K4me1, H3K27ac and H3K27me3 reads

mapped to each bin, respectively. We ordered the heatmap based on H3K4me1 reads intensity, and found that H3K27ac and H3K27me3 have distinct patterns. Then we conducted k-means clustering to cluster H3K4me1, H3K27ac and H3K27me3 peak sets into different categories. We tried multiple Ks and found k=5 generated best result. We then merged clusters with similar patterns. We have three categories of enhancers: H3k4me1 only, H3K4me1 + H3K27ac, H3K4me1 + H3K27me3.

We previously reported some promoters function as enhancers and regulate the expression level of other genes[8]. Those "enhancer-like" promoters usually have high H3K4me1 and low H3K4me3 enrichment. We identified "enhancer-like promoters" based on H4K4me1/H3K4me3 ratio around the promoter regions (+/- 2 kb around TSSs). We plotted density of the ratio and identified two peaks of the curve. We set 0 as the cutoff to separate promoters with "enhancer-like" properties. We separate protein-coding genes and lincRNA genes.

Identification of specific enhancers regulating upregulated and downregulated genes

We first compared genome-wide chromatin interaction network between HepG2 and THLE2 and identified specific enhancer-promoter interactions, we then identified specific enhancers regulating up- and down-regulated genes. In total we found there were 421 specific enhancers regulating 174 HCC specific (up-regulated) genes and 637 specific enhancers regulating HCC down-regulated genes.

Gene expression and enhancer number usage

Cell-specific genes in HepG2 and THLE2 were separated into three categories based on the number of connected enhancers: 1, 2 and >=3. Expression level for genes in each category was calculated and compared.

Transcription factor motif analysis

In order to characterize the sequence features of specific enhancers we preformed motif scanning using MEME suite[9]. We first narrowed down the candidate regions by using HepG2 ATAC-Seq peak regions downloaded from ENCODE Project (www.encodeproject.org). since open chromatin regions represented by ATAC-seq peaks are more likely bound by transcription factors. Then we extracted sequence regions using bedtools getfasta[1] and imported them into MEME.

ENCODE ChIP-Seq data analysis

Transcription factor ChIP-seq data (Bam format and peak file) for HepG2 cell line were downloaded from ENCODE data portal (https://www.encodeproject.org). All the TF binding sites were overlapped with cell-specific enhancers in both HepG2 and THLE2, and ranked by the number of enhancers they overlapped in HepG2. Top 20 TFs with the most number of overlapped HepG2 cell-specific enhancers were selected for signal intensity characterization. Normalized binding intensity (RPKM) were calculated for each TF around 2.5 kb regions of the submit of HepG2 cell-specific enhancers.

GWAS SNPs analysis

Liver related SNPs are downloaded from GWAS Catalog[10]. SNPs in linkage disequilibrium (LD SNPs, r2 >= 0.8) are selected based on HaploReg database V3[11]. All the SNPs were manually curated and are divided into "Liver cancer related" or "Normal liver related" SNPs and then overlapped with HepG2 and THLE2 cell-specific enhancers.

Whole genome sequence (WGS) data analysis

WGS data for both HepG2 and THLE2 were processed using Genome Analysis Toolkit (GATK)[12] . SNPs were called using GATK HaplotypeCaller.

Identification of A/B compartment

Hi-C data analysis has revealed that human genomes are organized into ~Mb base level compartment(Lieberman-Aiden et al., 2009), since CTCF associated ChIA-PET data can reach similar output as in situ Hi-C, we can also characterize A/B compartment using CTCF ChIA-PET data. Bam file with both ends uniquely mapped and redundancy removed were used to serve as an input to Juicer[13] 'Eigenvector' parameter, which extracts the first principle component (PCA1) of the Pearson's matrix[14]. Positive values were defined as A compartment and negative values were defined as B compartment. We used Juicer to identify compartments in 500 Kb resolution and compared compartments in HepG2 and THLE2. 2.73Gb genomic regions in HepG2 and 2.69 Gb genomic regions in THLE2 can be assigned to either A or B compartment.

Overall genome organization in HepG2 and THLE2

Human genomes are organized in a hierarchical manner. We were able to describe the comprehensive picture of multi-level organization combining CTCF and RNAPII ChIA-PET data, as well as the changes between HepG2 and THLE2. We can identify A/B compartment and CTCF Contact Domain (CCD) using CTCF data and RNAPII Associated Interaction Domain (RAID) using RNAPII data, enhancer-promoter interaction can also be

detected using RNAPII ChIA-PET data, thus a comprehensive genome organization mapped can be drawn.

Calculation of upregulated and downregulated genes in switched compartment

Compartment switch has been described in previous study[15] among different differentiation stages in human stem cell. Genomic regions (500Kb) were considered switched if compartments were flipped between THLE2 to HepG2 (A->B or B->A), and further were classified into four types based on compartment switch from THLE to HepG2: A->A, B->B, B->A and A->B. After we identified switched compartment from HepG2 and THLE2, we analyzed whether differentially expressed genes were associated with switched compartment. We defined that a gene is in a switched compartment if the promoter regions is overlapping with or is located with a flipped compartment. We found both up-regulated and down-regulated genes tended to stay in active compartment in both HepG2 and THLE2, this was reasonable since most of differentially expressed genes form large interaction complex with non-specific genes and active compartments were mostly determined by those active non-specific genes. Interestingly, for up-regulated genes in switched compartments, most of them were changed from repressed compartment in THLE2 to active compartment in HepG2 (103 gene in B->A compared with 11 genes in A->B), this was also the case for down-regulated genes (195 genes in A->B compared with 58 genes in B->A).

Comparison of CTCF binding peak

Cell specific and constitutive CTCF binding sites for HepG2 and THLE2 were identified using MAnorm[16] with the cutoff (p-value < 0.0001). Globally, we identified 40,955 constitutive peaks,15,993 specific HepG2 and 13,645 THLE2 specific peaks, respectively. We further dissect the CTCF peaks on the promoter regions (+/- 2 kb around TSSs) of upregulated and downregulated genes.

Calculation of CTCF binding breadth

CTCF binding sites in 99 ENCODE cell lines were downloaded from ENCODE portal (https://www.encodeproject.org) and CTCF binding breadth for specific CTCF peaks near HCC-specific and down-regulated genes were calculated.

Identification of CTCF anchor genes

HCC-specific and downregulated genes are classified as CTCF anchor and loops genes based on CTCF binding and connectivity. Genes within CCDs but not bound by CTCF are CTCF loop genes. Genes with CTCF binding and anchors are considered CTCF loop genes.

Normalization and visualization on browser

In-house BASIC Browser is used to visualize track-based view. Juicebox[13] is used for large-scale heatmap view.

RNA-seq bam files are normalized based on library size. Library with more reads are randomly downsampled into equal number of reads as the smaller library. ChIA-PET and ChIP-seq data are normalized based on sequence depth using deeptools[17].

Whole Genome Bisulfite Sequencing (WGBS) data analysis.

1. Quality control, alignment and methylation calling

R (R Core Team, 2016) version 3.3.2 was utilized for data analyses. Specific R packages and other tools/software used for analyses are noted in the respective sections below.The base quality of the fastq reads was checked using FastQC (version 0.11.5) (Andrews, S. Available at http://www.bioinformatics.babraham.ac.uk/projects/fastqc/) with default parameters. The raw reads were then aligned to bisulfite-converted human genome reference hg19 using bwa-meth (version 0.2.0)(Pedersen et al., 2014)with default parameters and produces alignments in SAM[18] format. Then the SAM alignments were converted in BAM format using Samtools 'view' function (-b, output in the BAM format). The aligned duplicated BAM reads were then marked and removed using BISCUIT (Available at https://github.com/zwdzwd/biscuit) 'markdup' function (-r, remove duplicates), and the methylation call at each CpG site was determined using MethylDackel (Available at https://github.com/dpryan79/MethylDackel) 'extract' function (--methylKit, output in the format required by methylKit).

2. Differentially methylated CpG sites (DMC) analysis

MethylKit (version 1.0.0)[19] was used for CpG site level quality control and DMC analysis. In order to do comparative analysis for quality control, all samples were first merged to one object for base-pair locations that are covered in all samples using 'unite' function with default parameters. The samples were then clustered based on the similarity of their methylation profiles using 'clusterSamples' function with default parameters.

CpG sites with at least 4x coverage annotated to CTCF motifs were considered for DMC analysis. In order to do DMC analysis, same as in quality control, all samples were then merged to one object for base-pair locations that are covered in all samples using 'unite' function with default parameters. The CpG sites with a methylation change smaller than 25% were filtered out to increase detection power[20]. The significance of differentially methylated CpG sites was determined using Fisher's Exact test, function 'calculateDiffMeth' with default parameters. For multiple-hypothesis testing, the significance cutoff was 0.05.

3. Differentially methylated motif analysis

MethylKit (version 1.0.0) was used for Differentially methylated motif analysis. CpG sites with at least 4x coverage annotated to CTCF motifs were considered for Differentially methylated motif analysis. The number of CpG sites overlapping with CTCF motifs was counted using 'regionCounts' function with default parameters.

All samples were then merged to one object for CpG site counts at CTCF motif regions that are covered in all samples using 'unite' function with default parameters. The CTCF motifs with a methylation change smaller than 25% were filtered out to increase detection power. The significance of differentially methylated CTCF motifs was determined using Fisher's Exact test, function 'calculateDiffMeth' with default parameters. For multiple-hypothesis testing, the significance cutoff was 0.05.

4. Integrative analysis

The association between CTCF peaks and CpG sites methylation levels was determined as follows. CpG sites with at least 4x coverage were considered for the integrative analysis. The statistical evaluation of the methylation level inside or outside of CTCF peaks and motifs was estimated using a Wilcoxon signed rank test (box-plots, ggplot2 version 2.2.1, H. Wickham, 2016). The significance cutoff was p value<0.05.

SUPPLEMENTAL REFERENCES

1 Quinlan, A. R. & Hall, I. M. BEDTools: a flexible suite of utilities for comparing genomic features. *Bioinformatics* **26**, 841-842, doi:10.1093/bioinformatics/btq033 (2010).

2 Bolger, A. M., Lohse, M. & Usadel, B. Trimmomatic: a flexible trimmer for Illumina sequence data. *Bioinformatics* **30**, 2114-2120, doi:10.1093/bioinformatics/btu170 (2014).

3 Dobin, A. *et al.* STAR: ultrafast universal RNA-seq aligner. *Bioinformatics* **29**, 15-21, doi:10.1093/bioinformatics/bts635 (2013).

4 Liao, Y., Smyth, G. K. & Shi, W. featureCounts: an efficient general purpose program for assigning sequence reads to genomic features. *Bioinformatics* **30**, 923-930, doi:10.1093/bioinformatics/btt656 (2014).

5 Love, M. I., Huber, W. & Anders, S. Moderated estimation of fold change and dispersion for RNA-seq data with DESeq2. *Genome Biol* **15**, 550, doi:10.1186/s13059-014-0550-8 (2014).

6 Li, H. & Durbin, R. Fast and accurate short read alignment with Burrows-Wheeler transform. *Bioinformatics* **25**, 1754-1760, doi:10.1093/bioinformatics/btp324 (2009).
7 Feng, J., Liu, T., Qin, B., Zhang, Y. & Liu, X. S. Identifying ChIP-seq enrichment using MACS. *Nat Protoc* **7**, 1728-1740, doi:10.1038/nprot.2012.101 (2012).
8 Li, G. *et al.* Extensive promoter-centered chromatin interactions provide a topological basis for transcription regulation. *Cell* **148**, 84-98, doi:10.1016/j.cell.2011.12.014 (2012).
9 Bailey, T. L. *et al.* MEME SUITE: tools for motif discovery and searching. *Nucleic Acids Res* **37**, W202-208, doi:10.1093/nar/gkp335 (2009).
10 Welter, D. *et al.* The NHGRI GWAS Catalog, a curated resource of SNP-trait associations. *Nucleic Acids Res* **42**, D1001-1006, doi:10.1093/nar/gkt1229 (2014).
11 Ward, L. D. & Kellis, M. HaploReg: a resource for exploring chromatin states, conservation, and regulatory motif alterations within sets of genetically linked variants. *Nucleic Acids Res* **40**, D930-934, doi:10.1093/nar/gkr917 (2012).
12 McKenna, A. *et al.* The Genome Analysis Toolkit: a MapReduce framework for analyzing next-generation DNA sequencing data. *Genome Res* **20**, 1297-1303, doi:10.1101/gr.107524.110 (2010).
13 Durand, N. C. *et al.* Juicer Provides a One-Click System for Analyzing Loop-Resolution Hi-C Experiments. *Cell Syst* **3**, 95-98, doi:10.1016/j.cels.2016.07.002 (2016).
14 Lieberman-Aiden, E. *et al.* Comprehensive mapping of long-range interactions reveals folding principles of the human genome. *Science* **326**, 289-293, doi:10.1126/science.1181369 (2009).
15 Dixon, J. R. *et al.* Chromatin architecture reorganization during stem cell differentiation. *Nature* **518**, 331-336, doi:10.1038/nature14222 (2015).
16 Shao, Z., Zhang, Y., Yuan, G. C., Orkin, S. H. & Waxman, D. J. MAnorm: a robust model for quantitative comparison of ChIP-Seq data sets. *Genome Biol* **13**, R16, doi:10.1186/gb-2012-13-3-r16 (2012).
17 Ramirez, F., Dundar, F., Diehl, S., Gruning, B. A. & Manke, T. deepTools: a flexible platform for exploring deep-sequencing data. *Nucleic Acids Res* **42**, W187-191, doi:10.1093/nar/gku365 (2014).
18 Li, H. *et al.* The Sequence Alignment/Map format and SAMtools. *Bioinformatics* **25**, 2078-2079, doi:10.1093/bioinformatics/btp352 (2009).
19 Akalin, A. *et al.* methylKit: a comprehensive R package for the analysis of genome-wide DNA methylation profiles. *Genome Biol* **13**, R87, doi:10.1186/gb-2012-13-10-r87 (2012).

20 Bourgon, R., Gentleman, R. & Huber, W. Independent filtering increases detection power for high-throughput experiments. *Proc Natl Acad Sci U S A* **107**, 9546-9551, doi:10.1073/pnas.0914005107 (2010).
